# Assessing medical professionalism: A systematic review of instruments and their measurement properties

**DOI:** 10.1371/journal.pone.0177321

**Published:** 2017-05-12

**Authors:** Honghe Li, Ning Ding, Yuanyuan Zhang, Yang Liu, Deliang Wen

**Affiliations:** 1 Research Center of Medical Education, China Medical University, Shenyang, Liaoning, China; 2 School of Public Health, Dalian Medical University, Dalian, Liaoning, China; 3 School of Public Health, China Medical University, Shenyang, Liaoning, China; Universita degli Studi di Firenze, ITALY

## Abstract

**Background:**

Over the last three decades, various instruments were developed and employed to assess medical professionalism, but their measurement properties have yet to be fully evaluated. This study aimed to systematically evaluate these instruments’ measurement properties and the methodological quality of their related studies within a universally acceptable standardized framework and then provide corresponding recommendations.

**Methods:**

A systematic search of the electronic databases PubMed, Web of Science, and PsycINFO was conducted to collect studies published from 1990–2015. After screening titles, abstracts, and full texts for eligibility, the articles included in this study were classified according to their respective instrument’s usage. A two-phase assessment was conducted: 1) methodological quality was assessed by following the COnsensus-based Standards for the selection of health status Measurement INstruments (COSMIN) checklist; and 2) the quality of measurement properties was assessed according to Terwee’s criteria. Results were integrated using *best-evidence synthesis* to look for recommendable instruments.

**Results:**

After screening 2,959 records, 74 instruments from 80 existing studies were included. The overall methodological quality of these studies was unsatisfactory, with reasons including but not limited to unknown missing data, inadequate sample sizes, and vague hypotheses. *Content validity*, *cross-cultural validity*, and *criterion validity* were either unreported or negative ratings in most studies. Based on *best-evidence synthesis*, three instruments were recommended: Hisar’s instrument for nursing students, Nurse Practitioners’ Roles and Competencies Scale, and Perceived Faculty Competency Inventory.

**Conclusion:**

Although instruments measuring medical professionalism are diverse, only a limited number of studies were methodologically sound. Future studies should give priority to systematically improving the performance of existing instruments and to longitudinal studies.

## Introduction

Facing medical professionals’ commitment to the society is being challenged by external forces of change within health care delivery systems, medical professionalism has received widespread attention as one of the core factors in providing high-quality patient care [1–4]. As demonstrated by many studies, professionalism is central to the practice of medicine because of its close associations with improvements in physician-patient relationships, patient satisfaction, health care professionals’ career satisfaction, and even healthcare outcomes [4–7]. The core components of medical professionalism require that all medical professionals commit to organize and deliver health care, to implement trust within patients and the public, and to self-monitor and improve in their respective fields [8–11]. Besides, understanding of professionalism varies across time and cultural contexts [12], suggesting that professionalism is a complex, multi-dimensional construct [9]. Therefore, for health researchers, educators and administrators, using and developing appropriate instruments to assess medical professionalism according to their purposes and target populations poses to be a challenge.

Over the last three decades, various instruments to assess medical professionalism were developed and employed in many empirical researches [13–15]. However, the validity of empirical findings is basically dependent on the quality of the instrument in use. Moreover, appropriate conclusions can only be drawn from high-quality assessment studies with proper measures. Therefore, selecting of an instrument carefully and based on the quality of instruments’ measurement properties was called for by many researchers[9, 16, 17].

In an effort to provide guidance for instrument usage, several published review articles have summarized and compared instruments assessing professionalism with respect to their content, type, and construction [9, 13, 15, 16, 18, 19]. These reviews have indicated that many instruments have not been fully evaluated for their measurement properties, which would then limit their usage [9, 13, 18]. To date, there is yet to be a systematic assessment of the quality of measurement properties of instruments measuring medical professionalism based on a universally accepted standardized framework.

The COnsensus-based Standards for the selection of health status Measurement INstruments (COSMIN) checklist is a widely accepted framework developed for systematically evaluating the methodological quality of studies [20–22] and has been used for assessing the quality of empirical studies in various fields [23–25]. Besides instruments measuring health care outcomes, the COSMIN checklist was also used to assess the quality of instruments of other complex health-related issues, such as self-efficacy, trust in physicians, and neighborhood environments [24, 26, 27]. A structured review of the different existing medical professionalism instruments and their performances can be able to facilitate the selection of an suitable instrument in accordance with the research purpose and target population. Moreover, this will help to understand the gaps and needs for further research.

In this study, by using the COSMIN checklist, we aimed 1) to summarize existing instruments for measuring medical professionalism and then to classify them according to their uses; 2) to assess the methodological quality of the studies examining the measurement properties of these instruments; 3) to evaluate the quality of identified instruments in terms of their measurement properties; and 4) to make recommendations for instrument selection based on *best-evidence synthesis* and to provide insights for future research.

## Materials and methods

### Search strategy

A systematic search of the electronic databases PubMed, Web of Science, and PsycINFO from January 1, 1990 through to December 31, 2015, was conducted to identify studies assessing medical professionalism with reports on measurement properties (S1 Appendix). Search strategy included a combination of the following five aspects in reference to the search construct developed by Terwee, et al. [28]: 1) construct search: professionalism AND 2) population search: physicians, residents, medical students, nurses, and nursing students AND 3) instruments AND 4) measurement properties AND 5) exclusion filter. The exclusion filter mainly limited publication types and subject groups according to Terwee’s criteria (S1 Appendix).

In this study, we identified professionalism to be a complete construct based on the classification of instruments by Arnold, et al. [29]. Arnold, et al., classified instruments assessing medical professionalism into three groups: those assessing professionalism as a facet of competence; those assessing professionalism as a comprehensive construct; and those assessing separate elements of professionalism, such as humanism and empathy [29]. This review included measures of professionalism as a comprehensive construct or as a facet of competency, since any measure of only an individual element of professionalism was not considered as a measure assessing professionalism as a whole.

In addition to the electronic database search, a secondary search was conducted by screening the references and citations of included full texts and of previous published reviews [9, 13, 15–19, 30], and then by searching using the names of the involved instruments.

### Study selection

Two researchers (LH and ZY) independently screened titles and abstracts of the included records for potential inclusion and independently evaluated full texts for eligibility by using the following inclusion criteria: 1) target population was physicians, residents, medical students, nurses, and nursing students, where the specialties of physicians and residents referenced the MeSH terms for “physicians” (https://www.ncbi.nlm.nih.gov/mesh/68010820); 2) English full text, articles in peer-reviewed journals, and original article; 3) described the development of an instrument or reported at least one or more measurement properties of the instrument; and 4) instrument assessed professionalism as a comprehensive construct or as a facet of competency.

Differences concerning inclusion criteria were resolved by means of discussion until a consensus was reached. If not, a third reviewer (DN) made the final decision.

### Data extraction and quality assessments

Before assessing the methodological quality of the included studies and the measurement properties of an instrument, descriptive variables of the included studies were extracted, including: the short name of the instrument, author/year, country, study design, target population, sample size, setting(s), age, and sex ratio. If an instrument did not have a specific short name in the study, a brief descriptive title using the first author’s last name was assigned. The descriptive variables of instruments contained total number of participants for each instrument, content of assessment, number of items, response options, administration method, generalizability (if applicable), the instrument’s domain, and the theoretical foundation of the instrument. Instruments were then classified and organized according to their usage in reference to Wilkinson, et al. [9] and Goldie’s [19] classification of instruments assessing medical professionalism, which has been widely accepted in this study field.

### Evaluation of methodological quality of the included studies

Methodological quality of the included studies was evaluated based on the COSMIN checklist [20]. The COSMIN checklist includes 9 boxes for classical test theory (CTT) based analyses (*internal consistency*, *reliability*, *measurement error*, *content validity*, *structural validity*, *hypothesis testing*, *cross-cultural validity*, *criterion validity*, and *responsiveness*) to rate different aspects of the design, methodological, and reporting quality of studies on instruments’ measurement properties. Each box contains 5 to 18 items measured on a 4-point scale (excellent, good, fair, or poor). For item response theory (IRT) models, there is only 1 box to rate its methodological quality. The lowest score for any item within the item determined the overall score for each box. *Cross-cultural validity* aimed to determine the performance of the items on a translated or culturally adapted instrument and whether or not the adapted instrument adequately reflects the performance of the items of the original version of the instrument. *Responsiveness* was defined by COSMIN as the ability of an instrument to detect change over time in the construct to be measured. A full description of the 9 measurement properties can be obtained from the COSMIN taxonomy [22]. The COSMIN checklist and the 4-point scale can be found on the COSMIN website [31].

### Evaluation of measurement properties of the included instruments

Extraction of all reported aspects of the measurement properties was performed according to the COSMIN checklist [20–22]. The measurement properties of the identified measures were evaluated based on the criteria for quality of measurement properties developed by Terwee et al [32] (as can be seen in Table 1), which have been used in many systematic reviews in different study fields [33–35]. The Terwee’s criteria can be applied to all 9 properties as listed in the COSMIN checklist. Each available property was rated as positive (“+”), indeterminate (“?”), or negative (“-”) depending on the rating of measurement properties for each study

**Table 1:** Terwee’s quality criteria for measurement properties [32].

Property	Rating	Quality Criteria
**Reliability**		
Internal consistency		
	+	Cronbach's alpha(s) ≥ 0.70
	?	Cronbach's alpha not determined or dimensionality unknown
	-	Cronbach's alpha(s) < 0.70
Reliability		
	+	ICC / weighted Kappa ≥ 0.70 OR Pearson’s r ≥ 0.80
	?	Neither ICC / weighted Kappa, nor Pearson’s r determined
	-	ICC / weighted Kappa < 0.70 OR Pearson’s r < 0.80
Measurement error		
	+	MIC > SDC OR MIC outside the LOA
	?	MIC not defined
	-	MIC ≤ SDC OR MIC equals or inside LOA
**Validity**		
Content validity		
	+	All items are considered to be relevant for the construct to be measured, for the target population, and for the purpose of the measurement AND the questionnaire is considered to be comprehensive
	?	Not enough information available
	-	Not all items are considered to be relevant for the construct to be measured, for the target population, and for the purpose of the measurement OR the questionnaire is considered not to be comprehensive
Structural validity		
	+	Factors should explain at least 50% of the variance
	?	Explained variance not mentioned
	-	Factors explain < 50% of the variance
Hypothesis testing		
	+	Correlations with instruments measuring the same construct ≥ 0.50 OR at least 75% of the results are in accordance with the hypotheses AND correlations with related constructs are higher than with unrelated constructs
	?	Solely correlations determined with unrelated constructs
	-	Correlations with instruments measuring the same construct < 0.50 OR < 75% of the results are in accordance with the hypotheses OR correlations with related constructs are lower than with unrelated constructs
Cross-cultural validity		
	+	No differences in factor structure OR no important DIF between language versions
	?	Multiple group factor analysis not applied AND DIF not assessed
	-	Differences in factor structure OR important DIF between language versions
Criterion validity		
	+	Convincing arguments that gold standard is “gold” AND correlation with gold standard ≥ 0.70
	?	No convincing arguments that gold standard is “gold”
	-	Correlation with gold standard < 0.70
**Responsiveness**		
Responsiveness		
	+	Correlation with changes on instruments measuring the same construct ≥ 0.50 OR at least 75% of the results are in accordance with the hypotheses OR AUC ≥ 0.70 AND correlations with changes in related constructs are higher than with unrelated constructs
	?	Solely correlations determined with unrelated constructs
	-	Correlations with changes on instruments measuring the same construct < 0.50 OR < 75% of the results are in accordance with the hypotheses OR AUC < 0.70 OR correlations with changes in related constructs are lower than with unrelated constructs

MIC = minimal important change; SDC = smallest detectable change; LoA = limits of agreement; ICC = intraclass correlation coefficient; DIF = differential item functioning; AUC = area under the curve

### Data synthesis and quality assessment

In order to determine instruments for recommendation for future use, best-evidence synthesis as proposed by the Cochrane Back Review Group [36, 37] was performed, with levels of instrument properties categorized as “strong”, “moderate”, “limited”, “conflicting”, or “unknown” (Table 2). The best-evidence synthesis combined three aspects for consideration: 1) the methodological quality of the measurement property stated by various studies, 2) the rating of the measurement properties of instruments, and 3) the number of studies for each instrument. For example, a measurement property of an instrument was rated as *strong positive* (“+++”) if multiple studies stated that the property had “good” methodological quality and a positive (“+”) rating OR if at least one study stated that the property had “excellent” methodological quality and a positive (“+”) rating. More rating rules can be seen in Table 2.

**Table 2:** Rating levels for the quality of a measurement property.

Level	Rating	Criteria
**Strong**	+++ or ---	Consistent findings in multiple studies of good methodological quality OR in one study of excellent methodological quality
**Moderate**	++ or --	Consistent findings in multiple studies of fair methodological quality OR in one study of good methodological quality
**Limited**	+ or -	One study of fair methodological quality
**Conflicting**	+/-	Conflicting findings
**Unknown**	?	Only studies of poor methodological quality

- = negative rating, + = positive rating,? = indeterminate rating

In addition to evidence synthesis, best-rated instruments were identified as those which had at least two *strong positive* (“+++”) or three *moderate positive* (“++”) properties and no *limited* or *negative* (“-”, “--” or “---”) measurement properties.

A duplicate assessment of the included studies was conducted by a second researcher to discuss or resolve any ambiguities ratings.

## Results

### Literature search and study selection

**Fig 1:**
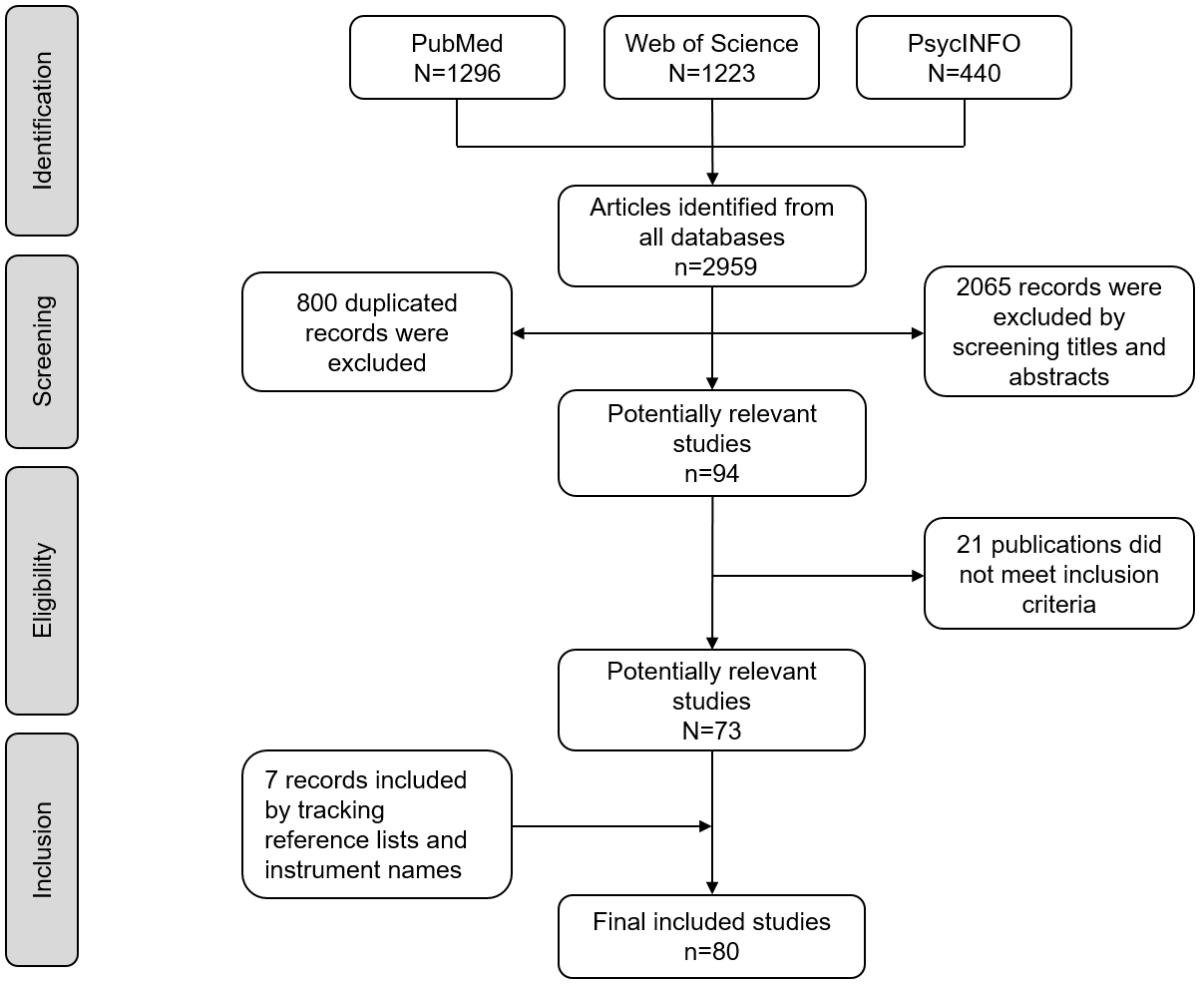
Flow diagram of the search and selection process.

The electronic database search of PubMed, Web of Science, and PsycINFO identified 2,959 total records. After screening titles and abstracts and excluding duplicated records, 94 studies were selected. Twenty-one of these failed to meet the inclusion criteria, mainly because they did not test the measurement properties of the instruments. Seven records that met the inclusion criteria were found through secondary search by screening the reference list of included publications and review articles. Ultimately, 80 research studies were included in this review. The details of the selection process can be seen in Fig 1.

### Description of included studies and instruments

The summary of the characteristics of the included studies (S2 Appendix) show that 78 of the 80 studies were published after 2000. More than 80% of studies were conducted in North America and Europe, including the United States, Canada, Netherlands, Spain, Turkey, and the United Kingdom. Except for 2 longitudinal studies from the United States and Netherlands, the rest were all cross-sectional studies. 37 studies developed new instruments. The number of participants in a study ranged from 12 [38] to 18,564 [39], with about 10% of the studies having less than 100 participants each.

A total of 74 instruments were divided into two broad categories depending on whether professionalism was recognized as a comprehensive construct (n = 44) or as a facet of competence (n = 30). And then the 80 included studies were divided according to the type of tools’ use of Wilkinson [9] and Goldie [19] taxonomy, instruments in each broad category were further classified into the following categories: self-administered rating, simulation, direct observation, multisource feedback (MSF), patients’ opinion, role model evaluation, and professionalism environment. The role mode evaluation category contained student or resident assessments of their instructor, clinical teacher, or faculties as a role model. The professionalism environment category contained studies assessing the medical professionalism of the practice or learning environment and not any specific individual. Among instruments regarding professionalism as a comprehensive construct, self-administered rating scales were most commonly used. In the category where professionalism was recognized as a facet of competency, MSF and direct observation were the most commonly used instrument. The classification of the 74 included instruments’ classification can be seen in Table 3, and details of the included instruments can be found in the S3 Appendix.

**Table 3:** Classification of instruments based on Wilkinson and Goldie taxonomy.

Type of tool use	Professionalism as a comprehensive construct		Professionalism as a facet for competency	
	Number of instrument	Number of study	Number of instrument	Number of study
**Self-administered rating**	14	17	5	4
**Simulation**	2	2	5	5
**Direct observation**	6	8	11	13
**Multi Source Feedback**	2	2	14	16
**Peer assessment**	1	1		
**Patients’ opinion**	1	1		
**Role model evaluation**	4	4	4	4
**Professionalism environment**	2	2	1	1

12 instruments were developed based on the theoretical framework of the American Board of Internal Medicine (ABIM) [3], 7 were based on the Royal College of Physicians and Surgeons of Canada (RCPSC) [40], and 22 were based on the Accreditation Council for Graduate Medical Education (ACGME) [41], accounting for 55.4% of all instruments. The rest of the instruments were constructed based on literature review or on qualitative analysis involving focus group discussions, the Delphi method, or interviews with experts. No IRT based study met the inclusion criteria.

### Methodological quality of the included studies

*Internal consistency* and *structural validity* were the most frequently reported measurement properties (reported in 64 and 54 studies, respectively), whereas *measurement errors*, *reliability*, *criterion validity* and *responsiveness* were not reported sufficiently, most likely due to the lack of follow-up studies (See Table 4). Inadequate sample sizes and lack of details in how missing data were managed resulted in 28 studies being rated as “fair” or “poor” in methodological quality. In 16 studies, each reported measurement property was rated as either “good” or “excellent”.

**Table 4:** Methodological quality of each study per measurement property.

Instrument	Authors/Year	Internal consistency	Reliability	Measurement error	Content validity	Structural validity	Hypothesis testing	Cross-cultural validity	Criterion validity	Responsiveness
**As a comprehensive construct**										
**Self-administered rating**										
Professionalism in Nursing Inventory	Miller/ 1993 [42]	Poor (3,7)	Fair (3)				Poor (3)			
Arnold scale (14-items)	Arnold / 1998 [43]	Good				Good	Poor (4)			
Arnold scale (12-items)	DeLisa/ 2001 [44]	Fair (3)				Fair (3)				
Arnold scale (17-items)	Aramesh/ 2009 [45]	Good				Good		Poor (14)		
PSCOM Professionalism Questionnaire	Blackall/ 2007 [46]	Good			Good	Good				
PSCOM Professionalism Questionnaire	Akhund/ 2014 [47]	Poor (5,6)					Fair (4)			
PSCOM Professionalism Questionnaire	Bustamante/ 2014 [48]	Excellent				Excellent		Good		
Tsai ABIM questionnaire	Tsai/ 2007 [49]	Poor (4,6)				Poor (4)				
Tsai ABIM questionnaire (Vietnamese)	Nhan/ 2014 [50]	Good				Good		Good		
Blue Multiple instruments	Blue/ 2009 [51]	Poor (7)				Poor (6)				
PSIQ	Crossley/ 2009 [52]					Poor*				
Hisar instrument for nursing students	Hisar/ 2010 [53]	Excellent	Good		Poor (2)	Excellent				
Jiang’s knowledge instrument	Jiang/ 2010 [54]	Good				Good				
LAMPS	Eraky/ 2013 [55]	Fair (3)								
Wittich Reflection instrument	Wittich/ 2013 [56]	Good				Good				
The new PAS	Ketis/ 2014 [57]	Good			Fair (4)	Good				
DUQuE professionalism instrument	Lombarts/ 2014 [58]	Good				Good	Fair (4)			
**Simulation**										
ECFMG-CSA	Zanten/ 2005 [59]						Good			
*p*-OSCE	Yang/ 2013 [60]		Good							
**Multi Source Feedback**										
GMC patient and colleague questionnaires	Campbell/ 2008 [39]	Poor (7)				Excellent				
*p*-360°evaluation	Yang/ 2013 [60]		Good							
**Direct observation**										
UMDSPAI	Gauger/ 2005 [61]	Poor (7)								
P-MEX	Cruess/ 2006 [62]					Good		Poor (14)		
P-MEX-Japanese version	Tsugawa/ 2009 [63]				Poor (4)	Fair (3)				
P-MEX-Japanese version 2	Tsugawa / 2011 [64]				Poor (4)	Good			Fair (4)	
EPRO-GP instrument	Camp/ 2006 [38]				Good					
Adaptation of AACS fro foreigner	Tromp/ 2007 [65]				Good					
Nijmegen Professionalism Scale	Tromp/ 2010 [66]	Poor (6)				Poor (4)				
*p*-mini-CEX	Yang/ 2013 [60]		Good							
**Peer assessment**										
Cottrell’s peer assessment	Cottrell/ 2006 [67]	Poor (5)								
**Patients’ opinion**										
Chandratilake’s general public scale	Chandratilake/ 2010 [68]	Poor (7)				Fair (3)				
**Role model evaluation**										
Ephgrave’s Assessment	Ephgrave/ 2006 [69]	Fair (4)				Poor (4)				
Arnold’s scale-environment version	Quaintance/ 2008 [70]	Poor (5)					Good			
LEP survey	Thrush/ 2011 [71]	Good				Good				
PACT	Young/ 2014 [72]	Poor (7)				Good				
**Professionalism environment**										
PEFWQ	Baumann/ 2009 [73]	Good	Good		Good	Good				
Gillespie’s scale	Gillespie/ 2009 [74]	Poor (5)			Good		Fair (4)			
**As one facet of competence**										
**Self-administered rating**										
Hotjat’s Jefferson competency scale	Hojat/ 2007 [75]	Fair (3)				Fair (3)	Poor (4)			
ABIM Patient Assessment	Symons/ 2009 [76]	Good				Good				
NPVS-R	Weis/ 2009 [77]	Fair (3)				Fair (3)				
NPVS-R	Lin/ 2010 [78]	Good			Poor (4)	Good				
VPPVS	Sang/ 2015 [79]	Good				Good				
NPRCS	Lin/ 2015 [80]	Excellent				Excellent				
**Multi Source Feedback**										
Musick 360-degree instrument	Musick/ 2003 [81]	Poor (5,7)								
Wood’s 360-degree evaluation	Wood/ 2004 [82]	Poor (5,7)								
CPSA-PAR MSF for anesthesiologists	Lockyer/ 2006 [83]	Poor (7)				Good				
CPSA-PAR MSF for emergency physicians	Lockyer/ 2006 [84]	Poor (7)				Good				
CPSA-PAR MSF for pediatricians	Violato/ 2006 [85]	Poor (7)				Poor (4)				
CPSA-PAR MSF for international doctors	Lockyer/ 2006 [86]	Poor (7)				Poor (4)				
CPSA-PAR MSF for Psychiatrists	Violato/ 2008 [87]	Poor (7)				Poor (4)				
CPSA-PAR MSF for physicians	Violato/ 2008 [88]	Poor (7)				Good				
CPSA-PAR MSF for P&LMP	Lockyer/ 2009 [89]	Poor (7)				Poor (4)				
CPSA-PAR MSF for Middle eastern interns	Ansari/ 2015 [90]	Poor (7)			Poor*	Poor (4)				
End-of-rotation evaluations	Park/ 2014 [91]						Good			
EOS group 360-degree instrument	Qu/ 2010 [92]	Poor (7)				Fair (3)		Poor*		
EOS group 360-degree instrument	Qu/ 2012 [93]	Poor (7)				Good				
EOS group 360-degree instrument	Zhao/ 2013 [94]	Poor (7)				Good				
Senol’s Turkish 360-degree assessment	Senol/ 2009 [95]	Poor (4,7)			Poor*					
Overeem’s MSF instruments	Overeem/ 2011 [96]	Good			Poor (4)	Good		Poor*		
**Direct observation**										
ACGME-TRF	Brasel/ 2004 [97]	Fair (3)			Poor*	Fair (3)				
Global rating form for ACGME competencies	Silber/ 2004 [98]	Good				Good				
ACGME general competencies	Reisdorff/ 2004 [99]					Poor (4)				
OCEX	Golnik/ 2004 [100]				Excellent					
OCEX	Golnik/ 2005 [101]	Poor (5)								
Durning’s Supervisor’s evaluation form	During/ 2005 [102]	Poor (7)				Good			Good	
Durning’s Supervisor’s evaluation form-PGY3	Artino/ 2015 [103]	Good				Good			Good	
Karayurt nursing students’ performance	Karayurt/ 2009 [104]	Good				Good				
COMPASS	Tromp/ 2012 [105]	Excellent			Fair (2)					Good
Handoff CEX-nurses	Horwitz/ 2013 [106]		Fair (3)				Fair (4)			
Handoff CEX-physicians	Horwitz/ 2013 [107]		Fair (3)				Fair (4)			
ITER	Kassam/ 2014 [108]	Fair (3)	Fair (2)			Fair (3)				
Dong’s Graduates Form	Dong/ 2015 [109]	Good				Good			Good	
**Simulation**										
SDOT	Shayne/ 2006 [110]		Good							
Jefferies’s OSCE of CanMEDS Roles	Jefferies/ 2007 [111]	Poor (6)					Poor (3)			
Carss’s Checklist of OSPRE	Carss/ 2011 [112]	Good				Poor (6)	Fair (4)		Fair (4)	
RO&CA	Musick/ 2010 [113]	Excellent				Excellent	Fair (4)			
ACGME competency checklist of OSCE	Yang/ 2011 [114]	Good				Good				
CanMEDS OSCE	Dwyer/ 2014 [115]	Poor (6)					Poor (3)			
**Role model evaluation**										
Smith’s instrument	Smith/ 2004 [110]	Good			Poor*	Poor (6)	Fair (4)			
Faculty Supervision Evaluation	Filho/ 2008 [116]	Poor (7)			Poor*	Good				
Colletti evaluation of clinical educators	Colletti/ 2010 [117]	Fair (3)				Fair (3)				
PFCI	Deemer/ 2011 [118]	Excellent				Excellent				
**Professionalism environment**										
MSSAPS	Liao/ 2014 [119]	Good				Good	Good			

PSCOM = The Penn State College of Medicine, PSIQ = Professional Self Identity Questionnaire, LAMPS = Learners’ Attitude of Medical Professionalism Scale, PAS = Professionalism Assessment Scale, DUQuE = Deepening Our Understanding of Quality Improvement in Europe, OSCE = Objective Structured Clinical Examination, ECFMG-CSA = Educational Commission for Foreign Medical Graduates’ clinical skills assessment, UMDSPAI = University of Michigan Department of Surgery Professionalism Assessment Instrument, P-MEX = Professionalism Mini-Evaluation Exercise, EPRO-GP = Evaluation of Professional Behavior in General Practice, AACS = Amsterdam Attitudes and Communications Scale, GMC = General Medical Council, PEFWQ = Factors in the Workplace Questionnaire, LEP = Learning environment for professionalism, PACT = The Professionalism Assessment of Clinical Teachers, MSSAPS = Medical Student Safety Attitudes and Professionalism Survey, NPVS-R = Nurses Professional Values Scale-Revised, VPPVS = Vietnamese Physician Professional Values Scale, NPRCS = Nurse Practitioners’ Roles and Competencies Scale, CPSA-PAR = The College of Physicians and Surgeons of Alberta, Physician Achievement Review, EOS = Education Outcomes Service Group, TRF = Traditional Rating Forms, PGY3 = Postgraduate Year 3, COMPASS = Competency Assessment List, OCEX = the Ophthalmic Clinical Evaluation Exercise, CEX = Clinical Evaluation Exercise, ITER = In-training Evaluation Report, OSPRE = Objective Structured Performance-Related Examination, RO&CA = Resident Observation and Competency Assessment, SDOT = Standardized Direct Observation Assessment Tool, PFCI = Perceived Faculty Competency Inventory

Numbers in parentheses for poor or fair ratings represent the item number in the respective COSMIN box.

* More than two items were assessed as “poor” level.

17 studies reported *content validity*, of which 11 were rated “fair” or “poor” in methodological quality because relevance or comprehensiveness was not sufficiently evaluated. 18 of the 71 studies implemented *hypothesis testing*, but only 4 were rated as “good”, and the rest failed to propose hypotheses or to clearly state hypothesis expectations (the directions or magnitudes of the effects). *Cross-culture validity* was tested for only five instruments, and poor performance in this property was mainly due to the lack of multiple-group confirmatory factor analysis. All but one of the 17 studies using MSF instruments performed poorly with respect to *internal consistency*, because Cronbach’s coefficients for subscales were not calculated.

### Quality of measurement properties

The quality of instruments’ measurement properties were assessed based on Terwee’s criteria [32] (Table 5). Most instruments performed well and were rated positively (“+”) in internal consistency and structural validity. Indeterminate results in *content validity* were mainly due to insufficient information. Due to the lack of multiple-group confirmatory factor analysis, most results for *cross-cultural validity* also returned indeterminate. As for *criterion validity*, there was insufficient evidence that the gold standards (i.e. USMLE, program GPA) used in two of the studies were in fact valid gold standards [97, 98]. Additionally, Pearson correlations between the instruments and these recognized gold standards were less than 0.7, signifying negative results. As a results, *criterion validity* displayed poor overall measurement performance.

**Table 5:** Summary of the measurement properties of instruments.

Instrument	Authors/Year	Internal consistency	Reliability	Measurement error	Content validity	Structural validity	Hypothesis testing	Cross-cultural validity	Criterion validity	Responsiveness
**As a comprehensive construct**										
**Self-administered rating**										
Professionalism in Nursing Inventory	Miller/ 1993 [42]	+	+				+			
Arnold scale (14-items)	Arnold / 1998 [43]	+				+	?			
Arnold scale (12-items)	DeLisa/ 2001 [44]	+				+				
Arnold scale (17-items)	Aramesh/ 2009 [45]	+				+		?		
PSCOM Professionalism Questionnaire	Blackall/ 2007 [46]	+			+	?				
	Akhund/ 2014 [47]	+					-			
	Bustamante/ 2014 [48]	+				+		-		
Tsai ABIM questionnaire	Tsai/ 2007 [49]	+				+				
	Nhan/ 2014 [50]	+				+		?		
Blue’s Multiple instruments	Blue/ 2009 [51]	-				?				
PSIQ	Crossley/ 2009 [52]					?				
Hisar’s instrument for nursing students	Hisar/ 2010 [53]	+	+		?	+				
Jiang’s knowledge instrument	Jiang/ 2010 [54]	+				-				
LAMPS	Eraky/ 2013 [55]	+								
Wittich Reflection instrument	Wittich/ 2013 [56]	+				?				
The new PAS	Ketis/ 2014 [57]	+			?	-				
DUQuE professionalism instrument	Lombarts/ 2014 [58]	?				?	+			
**Simulation**										
ECFMG-CSA	Zanten/ 2005 [59]	+					-			
*p*-OSCE	Yang/ 2013 [60]		+							
**Multi Source Feedback**										
GMC patient and colleague questionnaires	Campbell/ 2008 [39]	+				+				
*p*-360°evaluation	Yang/ 2013 [60]		+							
**Direct observation**										
UMDSPAI	Gauger/ 2005 [61]	+								
P-MEX	Cruess/ 2006 [62]					+		?		
	Tsugawa/ 2009 [63]				?	+				
	Tsugawa / 2011 [64]				?	+			?	
EPRO-GP instrument	Camp/ 2006 [38]				+					
Adaptation of AACS fro foreigner	Tromp/ 2007 [65]				+					
Nijmegen Professionalism Scale	Tromp/ 2010 [66]	+				+				
*p*-mini-CEX	Yang/ 2013 [60]		+							
**Peer assessment**										
Cottrell’s peer assessment	Cottrell/ 2006 [67]	+								
**Patients’ opinion**										
Chandratilake’s general public scale	Chandratilake/ 2010 [68]	+				?				
**Role model evaluation**										
Ephgrave’s Assessment	Ephgrave/ 2006 [69]	+				+				
Arnold’s scale-environment version	Quaintance/ 2008 [70]	+					+			
LEP survey	Thrush/ 2011 [71]	+				+				
PACT	Young/ 2014 [72]	+				+				
**Professionalism environment**										
PEFWQ	Baumann/ 2009 [73]	+	-		+	+				
Gillespie’s scale	Gillespie/ 2009 [74]	+			?		+			
**As one facet of competence**										
**Self-administered rating**										
Hotjat’s Jefferson competency scale	Hojat/ 2007 [75]	+				+	+			
ABIM Patient Assessment	Symons/ 2009 [76]	+				+				
NPVS-R	Weis/ 2009 [77]	+				+				
	Lin/ 2010 [78]	+			+	+				
VPPVS	Sang/ 2015 [79]	+				?				
NPRCS	Lin/ 2015 [80]	+				+				
**Multi Source Feedback**										
Musick 360-degree instrument	Musick/ 2003 [81]	+								
Wood’s 360-degree evaluation	Wood/ 2004 [82]	+								
CPSA-PAR MSF for anesthesiologists	Lockyer/ 2006 [83]	+				+				
CPSA-PAR MSF for emergency physicians	Lockyer/ 2006 [84]	+				+				
CPSA-PAR MSF for pediatricians	Violato/ 2006 [85]	+				+				
CPSA-PAR MSF for international doctors	Lockyer/ 2006 [86]	+				+				
CPSA-PAR MSF for Psychiatrists	Violato/ 2008 [87]	+				+				
CPSA-PAR MSF for physicians	Violato/ 2008 [88]	+				+				
CPSA-PAR MSF for P&LMP	Lockyer/ 2009 [89]	+				+				
CPSA-PAR MSF for Middle eastern interns	Ansari/ 2015 [90]	+			?	+				
End-of-rotation evaluations	Park/ 2014 [91]						+			
EOS group 360-degree instrument	Qu/ 2010 [92]	+				+		?		
	Qu/ 2012 [93]	+				+				
	Zhao/ 2013 [94]	+				+				
Senol’s Turkish 360-degree assessment	Senol/ 2009 [95]	+			?					
Overeem’s MSF instruments	Overeem/ 2011 [96]	+			+	+		?		
**Direct observation**										
ACGME-TRF	Brasel/ 2004 [97]	+			?	?				
Global rating form for ACGME competencies	Silber/ 2004 [98]	+				+				
OCEX	Golnik/ 2004 [100]				?					
	Golnik/ 2005 [101]	-								
ACGME general competencies	Reisdorff/ 2004 [99]					?				
Durning’s Supervisor’s evaluation form	During/ 2005 [102]	+				+				
Durning’s Supervisor’s evaluation form-PGY3	Artino/ 2015 [103]	+				+				
Karayurt nursing students’ performance	Karayurt/ 2009 [104]	+				+				
COMPASS	Tromp/ 2012 [105]	+			?					
Handoff CEX	Horwitz/ 2013 [106]		-				+			
	Horwitz/ 2013 [107]		+				-			
ITER	Kassam/ 2014 [108]	+	+			+				
Dong’s Graduates Form	Dong/ 2015 [109]	+				+				
**Simulation**										
SDOT	Shayne/ 2006 [110]		+							
Jefferies’s OSCE of CanMEDS Roles	Jefferies/ 2007 [111]	+					+			
Ponton-Carss Checklist of OSPRE	Carss/ 2011 [112]	-				?	+			
RO&CA	Musick/ 2010 [113]	+				?	+			
ACGME competency checklist of OSCE	Yang/ 2011 [114]	+				?				
CanMEDS OSCE	Dwyer/ 2014 [115]	+					+			
**Role Model evaluation**										
Smith instrument	Smith/ 2004 [110]	+	??		?	?	+			
Faculty Supervision Evaluation	Filho/ 2008 [116]	+			?	+				
Colletti evaluation of clinical educators	Colletti/ 2010 [117]	-				+				
PFCI	Deemer/ 2011 [118]	+				+				
**Professionalism environment**										
MSSAPS	Liao/ 2014 [119]	+				+	-			

### Best-evidence synthesis

*Best-evidence synthesis* was performed according to the method summarized in Table 2, by integrating the results of study methodological qualities (Table 4) and the results of measurement properties of instruments (Table 5). The performances of each instrument’s measurement properties are shown in Table 6. In general, instruments performed the best in *internal consistency* and *structure validity*, where 6 and 7 instruments achieved (“+++”) respectively. No study analyzed *measurement error*, and only one study reported on *responsiveness*. Among the studies reporting on *content validity* and the *cross-culture validity*, the majority of instruments received *indeterminate* (“?”) ratings, which means if the studies had poor methodological quality assessing the performance of these measurement properties, the exact performance of these measurement properties could not be determined irrespective of whether or not they were positively or negatively rated.

**Table 6:** Summary of best-evidence synthesis.

Target population	Instrument	Internal consistency	Reliability	Measurement error	Content validity	Structural validity	Hypothesis testing	Cross-cultural validity	Criterion validity	Responsiveness
**As a comprehensive construct**										
**Physicians**	**Self-administrated rating**									
	DUQuE professionalism instrument [58]	?				?	+			
	**Multi Source Feedback**									
	GMC patient and colleague questionnaires [39]	?				+++				
	**Patients’ opinion**									
	Chandratilake’s general public scale [68]	?				?				
**Residents**	**Self-administrated rating**									
	Arnold scale (14-items) [43]	++				++	?			
	Arnold scale (12-items) [44]	+				+				
	Arnold scale (17-items) [45]	++				++		?		
	Gillespie’s scale [74]	?			?		+			
	**Simulation**									
	ECFMG-CSA [59]						--			
	*p*-OSCE [60]	++								
	**Multi Source Feedback**									
	*p*-360°evaluation [60]	++								
	**Direct observation**									
	UMDSPAI[61]	?								
	P-MEX[62–64]				?	+++		?		
	EPRO-GP instrument[38]				++					
	Nijmegen Professionalism Scale [66]	?				?				
	Adaptation of AACS fro foreigner [65]				++					
	*p*-mini-CEX [60]	++								
	**Role model evaluation**									
	Ephgrave’s Assessment[69]	+				?				
	**Professionalism environment**									
	Gillespie’s scale [74]	?			?		+			
**Medical students**	**Self-administrated rating**									
	Arnold scale (14-items) [43]	++				++	?			
	PSCOM Professionalism Questionnaire [46–48]	+++			++	+++	-	--		
	Tsai ABIM questionnaire [49, 50]	++				++		?		
	PSIQ [52]					?				
	Blue’s Multiple instruments [51]	?				?				
	Jiang’s knowledge instrument [54]	++				--				
	LAMPS [55]	+								
	Wittich Reflection instrument [56]	++				?				
	The new PAS[57]	++			?	--				
	**Peer assessment**									
	Cottrell’s peer assessment [67]	?								
	**Role model evaluation**									
	Arnold’s scale-environment version [70]	?					++			
	PACT[72]	?				++				
	LEP survey[71]	++				++				
**Nurses**	**Self-administrated rating**									
	Professionalism in Nursing Inventory [42]	?	+				?			
	DUQuE professionalism instrument [58]	?				?	+			
**Nursing students**	**Self-administrated rating**									
	Hisar’s instrument for nursing students [53]	+++	++		?	+++				
	**Professionalism environment**									
	PEFWQ [73]	++	--		++	++				
**As one facet of competence**										
**Physicians**	**Self-administrated rating**									
	VPPVS [79]	++				?				
	**Multi Source Feedback**									
	CPSA-PAR MSF for anesthesiologists [83]	?				++				
	CPSA-PAR MSF for emergency physicians[84]	?				++				
	CPSA-PAR MSF for pediatricians [85]	?				?				
	CPSA-PAR MSF for Psychiatrists[87]	?				?				
	CPSA-PAR MSF for physicians [88]	?				++				
	CPSA-PAR MSF for P&LMP [89]	?				?				
	Overeem’s MSF instruments [96]	++			?	++		?		
	**Direct observation**									
	Handoff CEX[106, 107]		+/-				+/-			
**Residents**	**Self-administrated rating**									
	Hotjat’s Jefferson competency scale[75]	+				+	?			
	ABIM Patient Assessment-self assessment version [76]	++				++				
	**Multi Source Feedback**									
	Musick 360-degree instrument [81]	?								
	Wood’s 360-degree evaluation [82]	?								
	End-of-rotation evaluations [91]						++			
	EOS group 360-degree instrument[92–94]	?				+++		?		
	Senol’s Turkish 360-degree assessment [95]	?			?					
	CPSA-PAR MSF for international graduates [86]	?				?				
	**Direct observation**									
	ACGME-TRF [97]	+			?	?				
	Global rating form for ACGME competencies [98]	++				++				
	OCEX[100, 101]	?			++					
	ACGME general competencies [99]					?				
	Durning’s Supervisor’s evaluation form [102]	?				++			--	
	Durning’s Supervisor’s evaluation form-PGY3[103]	++				++			--	
	COMPASS [105]	+++			?					++
	ITER [108]	+	+			+				
	Dong’s Graduates Form [109]	++				++				
	**Simulation**									
	SDOT [110]		++							
	Jefferies’s OSCE of CanMEDS Roles [111]	?					?			
	Ponton-Carss Checklist of OSPRE [112]	--				?	+		-	
	RO&CA [113]	+++				?	+			
	ACGME competency checklist of OSCE[114]	++				?				
	CanMEDS OSCE [115]	?					?			
	**Role model evaluation**									
	Faculty Supervision Evaluation [116]	?			?	?				
	Colletti evaluation of clinical educators [117]	+				+				
	Smith instrument [120]	++			?	?	+			
**Medical students**	**Multi Source Feedback**									
	CPSA-PAR MSF for Middle eastern interns [90]	?			?	?				
	**Role model evaluation**									
	PFCI [118]	+++				+++				
	**Professionalism environment**									
	MSSAPS [119]	++				++	--			
**Nurses**	**Self-administrated rating**									
	NPVS-R [77, 78]	++			?	++				
	NPRCS [80]	+++				+++				
	**Direct observation**									
	Handoff CEX[106, 107]		+/-				+/-			
**Nursing students**	**Direct observation**									
	Karayurt nursing students’ performance [104]	++				++				

According to the data synthesis results, 3 instruments had at least two *strong positive* (“+++”) or three *moderate positive* (“++”) ratings without any *limited* or *negative* (“-”, “--” or “---”) ratings in measurement properties and were thus identified as best-rated. Two of these instruments, both self-administered rating scales in the nursing profession, were Hisar’s instrument for nursing students [53] and the Nurse Practitioners’ Roles and Competencies Scale (NPRCS) [80]. The third is the Perceived Faculty Competency Inventory (PFCI), a role model evaluation by medical students regarding medical professionalism as a facet of competency [118]. Further details on these 3 instruments and their respective studies can found in S2 and S3 Appendices.

## Discussion

A systematic search of the electronic databases PubMed, Web of Science, and PsycINFO was conducted to collect studies published from 1990–2015. 80 studies satisfied the inclusion criteria, and a total of 74 instruments for assessing medical professionalism were identified. The methodological quality of the studies and the instruments’ measurement properties were systematically evaluated according to the COSMIN checklist. The methodological qualities of studies were usually weakened by vague hypotheses, missing data, and inadequate sample sizes. The performances of instruments in *content validity*, *cross-cultural validity*, and *criterion validity* were unsatisfactory in most studies. Also, *measurement errors* and *responsiveness* were largely neglected by existing studies. Finally, based on *best-evidence synthesis*, three instruments were recommended: Hisar’s instrument for nursing students, the NPRCS, and the PFCI,.

Up and prior to 2009, several published articles systematically reviewed the assessment tools or techniques used to assess medical professionalism [9, 13, 15, 18]. However, recent systematic reviews mainly focus on a specific instrument type (eg. multisource feedback) or on a specific medical discipline [30, 121]. From 2009 onwards, there is yet to be a more up-to-date systematic review that comprehensively summarizes the existing instruments assessing medical professionalism, despite there being increasing attention and focus on the assessment of medical professionalism. In this review, we included new studies and a corresponding instrument published from 2008 to 2015, analyzes the methodological quality of the studies and the measurement properties of the reported instruments, and summarizes the instruments’ characteristics in order to facilitate their selection and use. Moreover, the COSMIN checklist was a critical appraisal tool for studying the quality of studies on instrument measurement properties. By using the COSMIN checklist to systematically assess and analyze each included study and its corresponding instrument, a summary on the performance of each instrument could be constructed based on a universally accepted standardized framework, which was not utilized in previous reviews.

The measurement instruments assessed in this review are diverse in target populations and tools’ uses. According to the type of tools’ uses [9], the instruments were divided into seven categories: self-administrated ratings, MSF, simulations (including OSCEs and high-fidelity patient simulations), patients’ opinions, direct observations (observed clinical encounters, such as min-CEX and P-MEX, and supervisor evaluations), role model evaluation, and professionalism environment. The last one is an additional category to Wilkinson’s classification of instruments assessing professionalism [9].

Direct observations (through mini-CEX and P-MEX) and collated views (through MSF and patients’ opinions) have been demonstrated to be crucial instruments for assessing professionalism [9, 122]. These offer different perspectives from multiple assessors and would enhance the breadth of assessment, reliability, and objectivity [9, 122]. However, despite there being 14 MSF instruments assessing professionalism as a facet of competency, this study showed that there were few MSF instruments assessing professionalism as a comprehensive concept. Furthermore, 17 of the 18 studies using MSF obtained a “poor” methodology rating for *internal consistency* or did not report on this property. Thus, there is a calling to refine and enhance the existing methodological quality of MSF instruments or to develop more MSF instruments specific to professionalism. Miller’s Taxonomy (knows, knows how, shows, and does) [123], as a template for the development of systems of evaluation [12, 124, 125], has often been used to illustrate the relative position and usage of assessment in medical education. The existing instruments assessing professionalism as a comprehensive construct also failed to demonstrate the “shows how” level of Miller’s pyramid model because of no simulation instruments, whereas assessment of professionalism as a facet of competency held better performance in this level.

Assessing professionalism usually implies the need to gather information to provide feedback, to guide remedial programs and decision-makers on grading, and to give referrals to promotion or certification decisions. However, in this study, very few of the involved instruments met the critical criteria for validity and reliability that would support their operational use for decision-making. Multiple previous reviews [9, 15, 18] have suggested that it may be more practical to improve the measurement properties of existing instruments rather than develop new measures of assessing medical professionalism. However, 37 of the instruments involved in this study were newly developed, and most of the existing instruments lacked refinement. In addition, good new instruments should be derived from sound qualitative research, repeated verification, and rigorous pilot studies [126]. In this review, few studies that developed a new instrument had good *content validity* (a crucial component in the development of a new instrument), demonstrated by failure to report details of how measurement items were derived. This limits the evidence available for developing and testing existing properties.

Both *reliability* and *measurement error* were ignored in many studies due to the lack of adequate follow-up. As can be seen in Tables 4, 5 and 6, based on the COSMIN definitions of measurement properties [22] and COSMIN checklist manual’s requirement of this measurement property [127], no study reported *measurement error*. It was defined as “the systematic and random error of a patient’s score that is not attributed to true changes in the construct to be measured” and needed to take into account the variance between time points. Thus, in this review none of the included studies reported acceptable *measurement error*. However, we also have to acknowledge that a large number of generalizability studies, especially those on direct observation instruments and MSF instruments, reported Standard Error Measurement (SEM). A possible explanation may be the difference between research assessments in medical education and healthcare outcome evaluations. Although medical education oriented assessments did not take the variance between time points into account to point out how the random error of the scores attribute to the true change, they instead used multiple evaluators to assess one target person to investigate the number of forms (evaluators) needed in order to obtain an estimate of the calculated average score via generalizability analysis. The generalizability coefficient reported by the included studies can be found in the “Administration/ generalizability” column of S3 Appendix. Thus, adjustment of the definition of *measurement error* in the COSMIN checklist would provide a better fit and also potentially include studies in the medical education context.

Lack of longitudinal studies and corresponding interventions are the primary reasons for the lack of evaluation of *responsiveness*. Additionally, *criterion validity* was also rarely reported. The most likely reason is that professionalism is an abstract concept. There is currently no universal definition of professionalism, not to mention a reasonable gold standard for its assessment. This is also the case in many other fields, such as trust in physicians [26], teamwork [128], communication skills [129, 130], and social skills [131].

After screening titles and abstracts, two IRT based studies assessing medical professionalism were found [133, 133]. However, they were not included in the review because they did not meet the inclusion criteria. Roberts *et al* only assessed the reasoning-skill of medical students, which was not a comprehensive concept of medical professionalism,[132] while another study did not include sociodemographic variables needed to assess differential item functioning [133]. This meant that it was not possible to obtain a total score for the methodological quality of these studies, since the assumptions for estimating parameters of the IRT model could not be checked. IRT models could provide more flexibility and has been widely used in medical education, especially for summative evaluation [134]. However, since it is a relatively modern theory, more evidence-based research is needed to confirm the applications and outcomes of IRT models in assessing medical professionalism.

As seen in the summary of *best-evidence synthesis*, no measurement instrument had been tested for all measurement properties, but three instruments—Hisar’s instrument for nursing students [53], the NPRCS [80], and the PFCI [118]—had better performance in both methodological quality and measurement properties. The former two self-administered rating scales belonged to the “knows” and “knows how” levels of Miller’s Taxonomy. This highlights the need for high-quality studies and for instruments that assess medical professionalism on higher cognitive levels of Miller’s Pyramid Model. Moreover, two of three recommended instruments assessed professionalism in nurses, while the third instrument targeted medical students. These could be referenced for the development or improvement of instruments assessing professionalism in other medical subfields, such as physicians.

The present review may be limited in its inclusion of studies and instruments. It is noted that there is also literature specific to each dimension of professionalism, such empathy, teamwork, lifelong learning, communication skills, or humanity. However, these do not represent professionalism as a whole. Therefore, studies of instruments specifically assessing these dimensions were not included in the search in order to maintain conceptual integrity. Researchers may wish to search for relevant instruments of specific concepts not included in this review. Furthermore, as with every systematic review, the results were limited by the inclusion criteria and the inclusion of only papers that were available as full text, and certain instruments for assessing professionalism may have been overlooked because the corresponding studies did not test for measurement properties.

## Conclusion

This study summarized and described 74 instruments for assessing medical professionalism from 80 existing studies and followed the COSMIN checklist to systematically evaluate these instruments’ measurement properties and the studies’ methodological quality. The instruments were diverse in tools’ use and target population, but the performance of their measurement properties and the methodological quality of the corresponding studies were varied. Specifically, *reliability* and *measurement error* were ignored in many studies due to the lack of adequate follow-up, and *responsiveness* was rarely reported due to lack of longitudinal study and corresponding intervention. For the measurement properties that were reported, *content validity* and *criterion validity* had more negative or indeterminate ratings, which would limit the usage of the instruments and the significance of assessment results. Thus, future studies should give priority to the application of existing instruments in different populations from various regions in order to verify the comparability of results based on these instruments. In addition, more follow-up investigations and longitudinal studies are needed. Of the instruments reviewed, Hisar’s instrument for nursing students, the Nursing Practitioner’s Roles and Competencies Scale, and Perceived Faculty Competency Inventory were best rated and had outstanding performance in both measurement properties and corresponding study methodological quality. However, there is still the need for high-quality instruments assessing medical professionalism in other subfields, such as for physicians. By taking the instruments’ performance and their type of tools’ use into account, we hope this review could help researchers or educators to choose suitable instruments according to their study purposes and target populations.

## Supporting information

S1 Appendix Search strategy for PubMed, Web of Science, and PsycINFO. (DOCX)

S2 Appendix Characteristics of included studies. (DOCX)

S3 Appendix Characteristics of included instruments. (DOCX)

S4 Appendix PRISMA 2009 checklist. (DOCX)
