# Supplementary material for: Assessing medical professionalism: A systematic review of instruments and their measurement properties
Source: PLoS One. 2017 May 12;12(5):e0177321. doi: 10.1371/journal.pone.0177321 (PMC5428933; doi:10.1371/journal.pone.0177321)
Supplement: S1 Appendix — (DOCX) [file pone.0177321.s001.docx]

**S1 Appendix. Search strategy for PubMed, Web of Science, and PsycINFO**

**[Q6, Q9] PubMed: 1296**

**Construct search:**

Professionalism[tiab] OR “professional performance”[tiab] OR “professional behavior”[tiab] OR “professional attitude”[tiab] OR (professional identity formation[tiab]) OR (professionalism[tiab] AND competenc*[tiab]) OR (professional AND competenc*[tiab])

**Population search:**

“Physicians”[Mesh] OR “Nurses”[Mesh] OR “Students, Medical”[Mesh] OR “Students, Nursing” [Mesh] OR Residents [tiab]

**Instrument search:**

Scale*[tiab] OR measure*[tiab] OR inventor*[tiab] OR questionnaire*[tiab] OR tool*[tiab] OR instrument*[tiab] OR assess*[tiab] OR evaluat*[tiab] OR indicator*[tiab] OR index*[tiab] OR survey[tiab] OR “self report”[tiab] OR test[tiab]

**Measurement properties:**

Instrumentation[sh] OR methods[sh] OR “Validation Studies”[pt] OR “Comparative Study”[pt] OR “psychometrics”[MeSH] OR psychometr*[tiab] OR clinimetr*[tw] OR clinometr*[tw] OR “outcome assessment (health care)”[MeSH] OR “outcome assessment”[tiab] OR “outcome measure*”[tw] OR “observer variation”[MeSH] OR “observer variation”[tiab] OR “Health Status Indicators”[Mesh] OR “reproducibility of results”[MeSH] OR reproducib*[tiab] OR “discriminant analysis”[MeSH] OR reliab*[tiab] OR unreliab*[tiab] OR valid*[tiab] OR coefficient[tiab] OR homogeneity[tiab] OR homogeneous[tiab] OR “internal consistency”[tiab] OR (cronbach*[tiab] AND (alpha[tiab] OR alphas[tiab])) OR (item[tiab] AND (correlation*[tiab] OR selection*[tiab] OR reduction*[tiab])) OR agreement[tiab] OR precision[tiab] OR imprecision[tiab] OR “precise values”[tiab] OR test-retest[tiab] OR (test[tiab] AND retest[tiab]) OR (reliab* [tiab] AND (test[tiab] OR retest[tiab])) OR stability[tiab] OR interrater[tiab] OR inter-rater[tiab] OR intrarater[tiab] OR intra-rater[tiab] OR intertester[tiab] OR inter-tester[tiab] OR intratester[tiab] OR intra-tester[tiab] OR interobserver[tiab] OR inter-observer[tiab] OR intraobserver[tiab] OR intraobserver[tiab] OR intertechnician[tiab] OR inter-technician[tiab] OR intratechnician[tiab] OR intra-technician[tiab] OR interexaminer[tiab] OR inter-examiner[tiab] OR intraexaminer[tiab] OR intra-examiner[tiab] OR interassay[tiab] OR inter-assay[tiab] OR intraassay[tiab] OR intra-assay[tiab] OR interindividual[tiab] OR inter-individual[tiab] OR intraindividual[tiab] OR intra-individual[tiab] OR interparticipant[tiab] OR inter-participant[tiab] OR intraparticipant[tiab] OR intra-participant[tiab] OR kappa[tiab] OR kappa’s[tiab] OR kappas[tiab] OR repeatab*[tiab] OR ((replicab*[tiab] OR repeated[tiab]) AND (measure[tiab] OR measures[tiab] OR findings[tiab] OR result[tiab] OR results[tiab] OR test[tiab] OR tests[tiab])) OR generaliza*[tiab] OR generalisa*[tiab] OR concordance[tiab] OR (intraclass[tiab] AND correlation*[tiab]) OR discriminative[tiab] OR “known group”[tiab] OR “factor analysis”[tiab] OR “factor analyses”[tiab] OR dimension*[tiab] OR subscale*[tiab] OR (multitrait[tiab] AND scaling[tiab] AND (analysis[tiab] OR analyses[tiab])) OR “item discriminant”[tiab] OR “interscale correlation*”[tiab] OR error[tiab] OR errors[tiab] OR “individual variability”[tiab] OR (variability[tiab] AND (analysis[tiab] OR values[tiab])) OR (uncertainty[tiab] AND (measurement[tiab] OR measuring[tiab])) OR “standard error of measurement”[tiab] OR sensitiv*[tiab] OR responsive*[tiab] OR ((minimal[tiab] OR minimally[tiab] OR clinical[tiab] OR clinically[tiab]) AND (important[tiab] OR significant[tiab] OR detectable[tiab]) AND (change[tiab] OR difference[tiab])) OR (small*[tiab] AND (real[tiab] OR detectable[tiab]) AND (change[tiab] OR difference[tiab])) OR “meaningful change” [tiab] OR “ceiling effect”[tiab] OR “floor effect”[tiab] OR “Item response model”[tiab] OR IRT[tiab] OR Rasch[tiab] OR “Differential item functioning”[tiab] OR DIF[tiab] OR “computer adaptive testing”[tiab] OR “item bank”[tiab] OR “cross-cultural equivalence”[tiab]

**Exclusion filter**

“addresses”[Publication Type] OR “biography”[Publication Type] OR “case reports”[Publication Type] OR “comment”[Publication Type] OR “directory”[Publication Type] OR “editorial”[Publication Type] OR “festschrift”[Publication Type] OR “interview”[Publication Type] OR “lectures”[Publication Type] OR “legal cases”[Publication Type] OR “legislation”[Publication Type] OR “letter”[Publication Type] OR “news”[Publication Type] OR “newspaper article”[Publication Type] OR “patient education handout”[Publication Type] OR “popular works”[Publication Type] OR “congresses” [Publication Type] OR “consensus development conference”[Publication Type] OR “consensus development conference, nih”[Publication Type] OR “practice guideline”[Publication Type]) NOT (“animals”[MeSH Terms] NOT “humans”[MeSH Terms]

Publication year: 1990.01.01-2015.12.31

**[Q6, Q9, Q11] Web of Science: 1223**

**Construct search:**

TS= (Professionalism OR “professional performance” OR “professional behavior” OR “professional attitude” OR (“professional identity formation”) OR (professionalism AND competenc*) OR (professional AND competenc*)

**Population search:**

TS=(physician* OR doctor* OR resident* OR clinician* OR "clinical practitioner*" OR "medical professional*" OR Allergists OR Anesthesiologists OR Cardiologists OR Dermatologists OR Endocrinologists OR “Foreign Medical Graduates” OR Gastroenterologists OR “General Practitioners” OR Geriatricians OR Nephrologists OR Neurologists OR Oncologists OR Ophthalmologists OR “Osteopathic Physicians” OR Otolaryngologists OR Pediatricians OR Physiatrists OR Pulmonologists OR Radiologists OR Rheumatologists OR Surgeons OR Urologists OR "medical student*" OR nurse* OR “nursing student*”)

**Instrument search:**

TS=(Scale* OR measure* OR inventor* OR questionnaire* OR tool* OR instrument* OR assess* OR evaluat* OR indicator* OR index* OR survey OR “self-report” OR test*)

**Measurement properties:**

TS=(psychometr* OR “discriminant analysis” OR reliab* OR unreliab* OR valid* OR coefficient OR homogeneity OR homogeneous OR “internal consistency” OR (cronbach* AND (alpha OR alphas)) OR (item AND (correlation* OR selection* OR reduction*)) OR agreement OR precision OR imprecision OR “precise values” OR test-retest OR (test AND retest) OR (reliab* AND (test OR retest)) OR stability OR interrater OR inter-rater OR intrarater OR intra-rater OR intertester OR inter-tester OR intratester OR intra-tester OR interobserver OR inter-observer OR intraobserver OR intraobserver OR intertechnician OR inter-technician OR intratechnician OR intra-technician OR interexaminer OR inter-examiner OR intraexaminer OR intra-examiner OR interassay OR inter-assay OR intraassay OR intra-assay OR interindividual OR inter-individual OR intraindividual OR intra-individual OR interparticipant OR inter-participant OR intraparticipant OR intra-participant OR kappa OR kappa’s OR kappas OR repeatab* OR ((replicab* OR repeated) AND (measure OR measures OR findings OR result OR results OR test OR tests)) OR generaliza* OR generalisa* OR concordance OR (intraclass AND correlation*) OR discriminative OR “known group” OR “factor analysis” OR “factor analyses” OR dimension* OR subscale* OR (multitrait AND scaling AND (analysis OR analyses)) OR “item discriminant” OR “interscale correlation*” OR error OR errors OR “individual variability” OR (variability AND (analysis OR values)) OR (uncertainty AND (measurement OR measuring)) OR “standard error of measurement” OR sensitiv* OR responsive* OR ((minimal OR minimally OR clinical OR clinically) AND (important OR significant OR detectable) AND (change OR difference)) OR (small* AND (real OR detectable) AND (change OR difference)) OR “meaningful change” OR “ceiling effect” OR “floor effect” OR “Item response model” OR IRT OR Rasch OR “Differential item functioning” OR DIF OR “computer adaptive testing” OR “item bank” OR “cross-cultural equivalence”)

**Limitation**

2000-2015; English; Article

**[Q6, Q9, Q11] PsycINFO: 440**

**Construct search:**

AB (Professionalism OR “professional performance” OR “professional behavior” OR “professional attitude” OR (“professional identity formation”) OR (professionalism AND competenc*) OR (professional AND competenc*))

**Population search:**

AB (physician* OR doctor* OR resident* OR clinician* OR "clinical practitioner*" OR "medical professional*" OR Allergists OR Anesthesiologists OR Cardiologists OR Dermatologists OR Endocrinologists OR “Foreign Medical Graduates” OR Gastroenterologists OR “General Practitioners” OR Geriatricians OR Nephrologists OR Neurologists OR Oncologists OR Ophthalmologists OR “Osteopathic Physicians” OR Otolaryngologists OR Pediatricians OR Physiatrists OR Pulmonologists OR Radiologists OR Rheumatologists OR Surgeons OR Urologists OR "medical student*" OR nurse* OR “nursing student*”)

**Instrument search:**

AB (Scale* OR measure* OR inventor* OR questionnaire* OR tool* OR instrument* OR assess* OR evaluat* OR indicator* OR index* OR survey OR “self-report” OR test*)

**Measurement properties:**

AB (psychometr* OR “discriminant analysis” OR reliab* OR unreliab* OR valid* OR coefficient OR homogeneity OR homogeneous OR “internal consistency” OR (cronbach* AND (alpha OR alphas)) OR (item AND (correlation* OR selection* OR reduction*)) OR agreement OR precision OR imprecision OR “precise values” OR test-retest OR (test AND retest) OR (reliab* AND (test OR retest)) OR stability OR interrater OR inter-rater OR intrarater OR intra-rater OR intertester OR inter-tester OR intratester OR intra-tester OR interobserver OR inter-observer OR intraobserver OR intraobserver OR intertechnician OR inter-technician OR intratechnician OR intra-technician OR interexaminer OR inter-examiner OR intraexaminer OR intra-examiner OR interassay OR inter-assay OR intraassay OR intra-assay OR interindividual OR inter-individual OR intraindividual OR intra-individual OR interparticipant OR inter-participant OR intraparticipant OR intra-participant OR kappa OR kappa’s OR kappas OR repeatab* OR ((replicab* OR repeated) AND (measure OR measures OR findings OR result OR results OR test OR tests)) OR generaliza* OR generalisa* OR concordance OR (intraclass AND correlation*) OR discriminative OR “known group” OR “factor analysis” OR “factor analyses” OR dimension* OR subscale* OR (multitrait AND scaling AND (analysis OR analyses)) OR “item discriminant” OR “interscale correlation*” OR error OR errors OR “individual variability” OR (variability AND (analysis OR values)) OR (uncertainty AND (measurement OR measuring)) OR “standard error of measurement” OR sensitiv* OR responsive* OR ((minimal OR minimally OR clinical OR clinically) AND (important OR significant OR detectable) AND (change OR difference)) OR (small* AND (real OR detectable) AND (change OR difference)) OR “meaningful change” OR “ceiling effect” OR “floor effect” OR “Item response model” OR IRT OR Rasch OR “Differential item functioning” OR DIF OR “computer adaptive testing” OR “item bank” OR “cross-cultural equivalence”)

Limiters - Publication Year: 1990-2015; Peer Reviewed; Publication Type: Peer Reviewed Journal; Population Group: Human

Narrow by Language: - English
