# Supplementary material for: Assessing medical professionalism: A systematic review of instruments and their measurement properties
Source: PLoS One. 2017 May 12;12(5):e0177321. doi: 10.1371/journal.pone.0177321 (PMC5428933; doi:10.1371/journal.pone.0177321)
Supplement: S2 Appendix — (DOCX) [file pone.0177321.s002.docx]

**[Q6, Q7, Q9, Q13]** S2 Appendix. Characteristics of included studies

| Instrument | Authors/Year | Country | Study design | Target population | Sample size (respond rate)* | Settings | Age (mean, SD) & sex ratio (female,%). |
| --- | --- | --- | --- | --- | --- | --- | --- |
| **As a comprehensive construct** | | | | | | | |
| **Self-administered rating** | | | | | | | |
| Professionalism in Nursing Inventory | Miller/ 1993 [42] | United States | Develop a new instrument | Registered nurses | 515 (32.2%) | By email | Age: 31-40  Female: 93.6% |
| Arnold’s scale (14-items) | Arnold / 1998 [43] | United States | Develop a new instrument | Medical students and residents | 565 (75%) | Hospitals (5 institutions) |  |
| Arnold’s scale (12-items) | DeLisa/ 2001 [44] | United States | Cross-sectional study | Residents | 72 (59%) | Hospitals (6 institutions) |  |
| Arnold’s scale (17-items) | Aramesh/ 2009 [45] | Iran | Cross-sectional study | Residents | 272 (96.4%) | Two medical universities |  |
| PSCOM Professionalism Questionnaire | Blackall/ 2007 [46] | United States | Develop a new instrument | Medical tudents | 765 (51%) |  | Female: 44% |
| PSCOM Professionalism Questionnaire | Akhund/ 2014 [47] | Pakistan | Cross-sectional study | Undergraduate medical students | 127 (94%) | International medical college | Age: 21.11 ± 2.72  Female: 56.7% |
| PSCOM Professionalism Questionnaire | Bustamante/ 2014 [48] | Spain | Cross-sectional study (cross-culture) | Medical students | 250 (87%) | Medical school |  |
| Tsai ABIM questionnaire | Tsai/ 2007 [49] | China Taiwan | Develop a new instrument | Medical students | 133 | University and hospital | Age: 25.38 (24-27)  Female: 25% |
| Tsai ABIM questionnaire (Vietnamese) | Nhan/ 2014 [50] | Vietnam | Cross-sectional study | Medical students | 1028 (85.95%) | A Medical school | Female: 44.5%; Age: 21.6 |
| Blue’s Multiple instruments | Blue/ 2009 [51] | United States | Cross-sectional study | Medical students | 666 | Two Medical schools |  |
| PSIQ | Crossley/ 2009 [52] | Untied Kingdom | Cross-sectional study | Medical students | 496 | Medical school |  |
| Hisar’s instrument for nursing students | Hisar/ 2010 [53] | Turkey | Develop a new instrument | Final-year undergraduate nursing students | 1339 (80.2%) | 23 medical schools | Female: 88.3% |
| Jiang’s knowledge instrument | Jiang/ 2010 [54] | China | Develop a new instrument | First-semester medical students | 973 (50%) for open-ended survey; 1730 (88%) for close-ended survey | A University |  |
| LAMPS | Eraky/ 2013 [55] | Netherlands | Develop a new instrument | Medial students | 413 | Medial schools in Egypt and Saudi Arabia |  |
| Wittich Reflection instrument | Wittich/ 2013 [56] | United States | Develop a new instrument | Medical students | 161 (90.4%) | Two Medical schools |  |
| The new PAS | Ketis/ 2014 [57] | Slovenia | Develop a new instrument | Medical students | 112 (27.2%) | A Medical school | Female: 77.9%; Age: 22.1 ± 2.1 |
| 18  DUQuE professionalism instrument | Lombarts/ 2014 [58] | Netherlands | Develop a new instrument | Physicians and registered nurses | 2067 (69.8%) physicians and 2805 (94.8%) nurses | 74 hospitals in 7 European countries | Female: 67.9%; Age: 39.2 ± 9.7 |
| **Simulation** | | | | | | | |
| ECFMG^®^-CSA^®^ | Zanten/ 2005 [59] | United States | Cross-sectional study | Graduates from international medical schools | 7746 first-time takers | ECFMG | Age: 30.5±5.3  Female: 42% |
| *p*-OSCE | Yang/ 2013 [60] | China Taiwan | Cross-sectional study | Postgraduate first-year residents | 189 | A teaching hospital |  |
| **Multi Source Feedback** |  |  |  |  |  |  |  |
| *p*-360°evaluation | Yang/ 2013 [60] | China Taiwan | Cross-sectional study | Postgraduate first-year residents | 189 | A teaching hospital |  |
| GMC patient and colleague questionnaire | Campbell/ 2008 [39] | United Kingdom | Cross-sectional study | 1.5 Physicians, about 33 patients, and 12 colleagues for each physicians | 541 physician, 13754 patients for 380 doctors, and, 4269 colleagues of 309 doctors | Cross the national |  |
| **Direct observation** | | | | | | | |
| UMDSPAI | Gauger/ 2005 [61] | United States | Develop a new instrument | Residents | 103 faculty surveys and 45 self surveys |  |  |
| P-MEX | Cruess/ 2006 [62] | Canada | Develop a new instrument | Undergraduate medical students | 211 forms were completed on 74 students by 47 evaluators (faculties) |  |  |
| P-MEX-Japanese version | Tsugawa/ 2009 [63] | Japan | Cross-sectional study | Residents | 184 forms were completed on 23 senior residents by 41 evaluators (faculty members, peer residents, non-doctor professionals and junior residents) | A teaching hospital | Age: 29 (26-36)  Female: 22% |
| P-MEX-Japanese version 2 | Tsugawa / 2011 [64] | Japan | Cross-sectional study | Residents | 837 forms from 453 attending physicians, 165 nurses, 96 peers, and 123 junior residents assessed 165 residents. | Seven hospitals |  |
| EPRO-GP instrument | Camp/ 2006 [38] | Netherlands | Develop a new instrument | Postgraduate GP trainees and their GP trainers | 12 couples |  |  |
| Nijmegen Professionalism Scale | Tromp/ 2007 [65] | Netherlands | Cross-sectional study | General practitioner trainees | 116 (97.5%) GP trainers and 116 (97.5%)GP trainees | A Medical Center |  |
| Adaptation of AACS fro foreigner | Tromp/ 2010 [66] | Netherlands | Develop a new instrument | Foreign medical graduates | 28 supervisors, 8 nurses and 9 residents judged the 28 foreign medical graduates, finally has 54 evaluations | Two medical school |  |
| *P*-mini-CEX | Yang/ 2013 [60] | China Taiwan | Cross-sectional study | Postgraduate first-year residents | 189 | A teaching hospital |  |
| **Peer assessment** | | | | | | | |
| Cottrell’s peer assessment | Cottrell/ 2006 [67] | United States | Develop a new instrument | First-year medical students | 111 medical students, about 7 ratings for each students (91%) | A Medical School |  |
| **Patients’ opinion** | | | | | | | |
| Chandratilake’s general public scale | Chandratilake/ 2010 [68] | Scotland | Cross-sectional study | General public assess physicians | 953 patients | Online |  |
| **Role model evaluation** | | | | | | | |
| Ephgrave’s Assessment | Ephgrave/ 2006 [69] | United States | Develop a new instrument | Faculties (evaluated by residents) | 32 evaluations from 16 surgical residents, and 37 evaluations from 19 pediatrics residents | One institution |  |
| Arnold’s scale-environment version | Quaintance/ 2008 [70] | United States | Cross-sectional study | Medical students and faculties | Students: 376 (60%); faculties: 28 | Three Medical schools | Female: 49%; Age: 25-34 |
| LEP survey | Thrush/ 2011 [71] | United States | Develop a new instrument | tudents assess residents and attending physicians | 903 (81.3%) | Medical University’s affiliated hospitals |  |
| PACT | Young/ 2014 [72] | Canada | Develop a new instrument | Medical students assess Clinical teachers | 178 third-year clerk assess 567 faculties with 4715 evaluations |  |  |
| **Professionalism environment** | | | | | | | |
| PEFWQ^®^ | Baumann/ 2009 [73] | Canada | Develop a new instrument | Nurses | 848 for pilot study and 111 for re-test | Hospitals | Age: 44.6±10.65  Female: 94.9% |
| Gillespie’s scale | Gillespie/ 2009 [74] | United States | Cross-sectional study | Residents | 85 (77%) | Medical University’s affiliated hospital |  |
| **As one facet of competence** | | | | | | | |
| **Self-administered rating** | | | | | | | |
| Hotjat’s Jefferson assessment of competency | Hojat/ 2007 [75] | United States | A longitudinal study | Residents doctors graduated between 1975 and 2004 | 4560 | 508 hospitals | Female: 27.85% |
| ABIM Patient Assessment -self assessment version | Symons/ 2009 [76] | United States | Cross-sectional study | Residents | 130 (87.2%) | Medical University’s affiliated hospitals | Female: 60.8% |
| NPVS-R | Weis/ 2009 [77] | United States | Cross-sectional study | Nurses | 782 nursing students, graduating nursing students, and practicing nurses | Hospitals | Female: 91% |
| NPVS-R | Lin/ 2010 [78] | China Taiwan | Cross-sectional study | Nurses | 333 nursing students | A medical University |  |
| VPPVS | Sang/ 2015 [79] | Vietnam | Development | Physicians | 1086 | 5 hospitals | Female: 43.6%; Age: 41 |
| NPRCS | Lin/ 2015 [80] | China Taiwan | Cross-sectional study | Nurse practitioners | 563 (94.5%) | Multiple Hospitals | Female: 99.1%; Age: 35.1 ±4.8 |
| **Multi Source Feedback** | | | | | | | |
| Musick’s 360-degree instrument | Musick/ 2003 [81] | United States | Develop a new instrument | Residents in acute inpatient rehabilitation settings | Residents: 18; evaluations from therapists, nurses, social workers, case managers, and psychologists: 421 |  | Female: 27.8% |
| Wood’s 360-degree evaluation | Wood/ 2004 [82] | United States | Cross-sectional study | Radiology residents (residents, faculty, and patients) | 7 residents with 57 (57%) residents-patients interactions | A Hospital |  |
| CPSA-PAR MSF for anesthesiologists | Lockyer/ 2006 [83] | Canada | Develop a new instrument | Anesthesiologists | 186 anesthesiologists, 3135 patient surveys, 1415 coworker surveys, 1407 medical colleague surveys, 182 self surveys | A private company recruited the physicians |  |
| CPSA-PAR MSF for emergency physicians | Lockyer/ 2006 [84] | Canada | Develop a new instrument | Emergency physicians | 187 self-surveys, 1430 colleague surveys, 4039 patient surveys, and 1420 coworker surveys. | A private company recruited the physicians |  |
| CPSA-PAR MSF for pediatricians | Violato/ 2006 [85] | Canada | Develop a new instrument | Pediatricians | 100 self surveys, 2341 patient surveys, 758 coworker surveys, and 764 medical colleague surveys | A private company recruited the physicians |  |
| CPSA-PAR MSF for international doctors | Lockyer/ 2006 [86] | Canada | Develop a new instrument | International medical graduates | 34 self surveys, 647 patient surveys, 248 coworker surveys, and 201 medical colleague surveys | Paper-based survey in hospital | Female: 26.5% |
| CPSA-PAR MSF for Psychiatrists | Violato/ 2008 [87] | Canada | Develop a new instrument | Psychiatrists | 101 self surveys, 2456 patient surveys, 744 coworker surveys, and 764 medical colleague surveys | A private company recruited the physicians |  |
| CPSA-PAR MSF for physicians | Violato/ 2008 [88] | Canada | 5-year longitudinal study | Physicians | Doctors: 250; medical colleagues: Doctors*7.19 for first time and 7.65 for second time; co-workers: doctors*7.34 for first time and 7.61 for second time; patients: doctors*24.09 for first time and 24.39 for second time | A private company recruited the physicians | Female: 32.4% |
| CPSA-PAR MSF for Pathologists/Laboratory Medicine Physicians | Lockyer/ 2009 [89] | Canada | Cross-sectional study | Pathologists/Laboratory Medicine Physicians | 101 participants, 738 peer surveys, 730 referring physicians surveys, 742 coworker surveys | Hospitals |  |
| CPSA-PAR MSF for Middle eastern interns | Ansari/ 2015 [90] | Bahrain | Cross-sectional study (Cross-culture) | Interns | 21 interns, 116 senior interns surveys, 105 coworker surveys, and 93 medical colleague surveys | Teaching hospital | Female: 57.1% |
| End-of-rotation evaluation | Park/ 2014 [91] | United States | Cross-sectional study | Residents | 4986 evaluations of  291 internal medicine residents fro fellows, faculties, and program directors |  |  |
| EOS group 360-degree instrument | Qu/ 2010 [92] | China | Cross-sectional study | Residents physicians | 148 residents, no information for other evaluators | Hospital (Multicenter study) |  |
| EOS group 360-degree instrument | Qu/ 2012 [93] | China | Cross-sectional study | Residents physicians | 258 self surveys, 258 attending physician surveys, 744 nurse surveys, 1806 patients surveys, 516 peers surveys, 516 office staff surveys | Hospital (Multicenter study) |  |
| EOS group 360-degree instrument | Zhao/ 2013 [94] | China | Cross-sectional study | Surgery Residents | 149 Surgery Resident surveys; 149 attending physician surveys, 447 nurse surveys; 1043 patient surveys; 298 peer surveys, and 298 office staff surveys | Hospital (Multicenter study) |  |
| Senol’s Turkish 360-degree assessment | Senol/ 2009 [95] | Turkey | Develop a new instrument | Dermatology residents | 7 residents evaluated by 259 raters (residents, faculties, nurses, auxiliary staffs, secretaries and patients) | A Hospital |  |
| Overeem’s MSF instruments | Overeem/ 2011 [96] | Netherlands | Develop a new instrument | Physicians | 146 hospital-based physicians with Ratings of 864 peers, 894 co-workers and 1960 patients | Hospitals | Female: 40% |
| **Direct observation** | | | | | | | |
| ACGME-TRF | Brasel/ 2004 [97] | United States | Cross-sectional study | Surgical residents | Surgical residents: 36; evaluations from 74 faculties: 343 |  |  |
| Global rating form for ACGME competencies | Silber/ 2004 [98] | United States | Cross-sectional study | Residents | 1295 (95%) | By mail and telephone; hospital and medical center | Female: 37% |
| OCEX | Reisdorff/ 2004 [99] | United States | Cross-sectional study | Ophthalmic Residents |  |  |  |
| OCEX | Golnik/ 2004 [100] | United States | Cross-sectional study | Ophthalmic Residents |  |  |  |
| ACGME general competencies | Golnik/ 2005 [101] | United States | Cross-sectional study | Emergency medicine resident | 5 resident program directors and 150 residents |  |  |
| Durning’s Supervisor’s evaluation form | During/ 2005 [102] | United States | Develop a new instrument | First year Residents | Residents: 1559 (80%), 1247 evaluations from supervisors | A University affiliated hospital |  |
| Durning’s Supervisor’s evaluation form-PGY3 | Artino/ 2015 [103] | United States | Cross-sectional study | Third year residents | 388 (76%) forms | A University affiliated hospital |  |
| Karayurt nursing students’ performance | Karayurt/ 2009 [104] | Turkey | Develop a new instrument | Nursing students | 97 students and 350 evaluations | A medical school | All female |
| COMPASS | Tromp/ 2012 [105] | Netherlands | Longitudinal study (one time per 1 month in three monhts) | First year postgraduate students (residents) | 68 (91.9%) GPs with 184 lists (forms) | A medical center |  |
| Handoff CEX-nurse | Horwitz/ 2013 [106] | United States | Develop a new instrument | Nurses | 25 shift-to-shift nurse reports yielded a total of 49+49 evaluations (similar with mini-CEX) | A Hospital |  |
| Handoff CEX-physicians | Horwitz/ 2013 [107] | United States | Develop a new instrument | Physicians | 97 physicians had 673 evaluations | Two University affiliated hospitals |  |
| ITER | Kassam/ 2014 [108] | Canada | Cross-sectional study | Residents | 172 | A teaching hospital |  |
| Dong’s Graduates Form | Dong/ 2015 [109] | United States | Develop a new instrument | Residents | 293 (86.2) residents | A teaching hospital |  |
| **Simulation** | | | | | | | |
| SDOT | Shayne/ 2006 [110] | United States | Cross-sectional study | Emergency medicine residents | 82 faculties assess two videos | A medical school |  |
| Jefferies’s OSCE of CanMEDS Roles | Jefferies/ 2007 [111] | Canada | Cross-sectional study | Candidate (residents) | 24 candidates and 13 examiners | University |  |
| Ponton-Carss Checklist of OSPRE | Carss/ 2011 [112] | United States | Cross-sectional study | Residents | 14 general surgery residents, 19 raters in 7 stations, thus had 89 forms | A Medical school | Female: 20% |
| RO&CA | Musick/ 2010 [113] | United States | Cross-sectional study | Residents | 362 (91.4%) evaluations were completed on 88 residents | Hospital (Multicenter study) |  |
| ACGME competency checklist of OSCE | Yang/ 2011 [114] | China Taiwan | Cross-sectional study | First year residents | 192 resident has 6 stations | A Teaching hospital |  |
| CanMEDS OSCE | Dwyer/ 2014 [115] | Canada | Cross-sectional study | Residents | 25 residents has 25*6=150 evaluations | A medical school |  |
| **Role model evaluation** | | | | | | | |
| Smith’s instrument | Smith/ 2004 [110] | United States | Develop a new instrument | Inpatient Attending Physicians | Attending physicians: 99; 145 residents completed 731 evaluations | A University affiliated public hospital |  |
| Faculty Supervision Evaluation | Filho/ 2008 [116] | Brazil | Develop a new instrument | Faculty | 19 residents, 39 instructors, 970 evaluations | A University affiliated public hospital |  |
| Colletti evaluation of clinical educators | Colletti/ 2010 [117] | United States | Develop a new instrument | Clinical educators | 29 faculties to assess two video of residents’ performance | A hospital |  |
| PFCI | Deemer/ 2011 [118] | United States | Develop a new instrument | Faculty | 88 doctoral students | A hospital | Female: 81.8% |
| **Professionalism environment** | | | | | | | |
| MSSAPS | Liao/ 2014 [119] | United States | Develop a new instrument | Fourth-year medical students | 228 (62%) |  |  |

* If the study did not reported the respond rate, we will let the parentheses blanket.
