# Supplementary material for: Assessing medical professionalism: A systematic review of instruments and their measurement properties
Source: PLoS One. 2017 May 12;12(5):e0177321. doi: 10.1371/journal.pone.0177321 (PMC5428933; doi:10.1371/journal.pone.0177321)
Supplement: S3 Appendix — (DOCX) [file pone.0177321.s003.docx]

**[Q6, Q7, Q9, Q13]** S3 Appendix. Characteristics of included instruments

| Instrument | Total participants | Content of assessing | Number of items | Response options | Administration/ Generalizability * | Instrument’s domain | Theory foundation |
| --- | --- | --- | --- | --- | --- | --- | --- |
| **Professionalism as a comprehensive construct** | | | | | | | |
| **Self-administered rating** | | | | | | | |
| Professionalism in Nursing Inventory [42] | 515 | Professionalism behavior | 9 | Dichotomous measure of yes or no (0 to 1) | Self administration | Educational background, adherence to the code of ethics, participation in the professional organization, continuing education and competency, communication and publication, autonomy and self-regulation, community service, theory use, development, and evaluation, and research involvement. | Literature review and Miller’s model for professionalism |
| Arnold scale (14-items) [43] | 909 | Attitude and behavior in medical education and residency training environment | 14 | A 10-point Likert scale (10=most positive respond) | Self administration | Excellence, honor/integrity, altruism/respect | Professionalism defined by American Board of Internal Medicine (ABIM)[116] |
| Arnold scale (12-items) [44] |  |  | 12 |  |  |  |  |
| Arnold scale (17-items) [45] |  |  | 17 |  |  |  |  |
| PSCOM Professionalism Questionnaire [46-48] | 1142 | Attitudes toward professionalism in medical education among students, or residents and faculty | 36 | A five-point Likert scale: 1=never, 5=great deal) and A five-point Likert scale: 1=highest rank, 5=lowest rank) | Self administration | Accountability, enrichment, equity, honor& integrity, altruism, duty, and respect | ABIM |
| Tsai ABIM questionnaire [49, 50] | 1161 | Structure of ‘latent traits’ underlying the concept of medical professionalism | 32 | A 5-point Likert scale (1=Not important at all, 5= Very important) | Self administration | Commitment to care; righteous and rule abiding; pursuing quality patient care; habit of professional practice; interpersonal relationship; patient-oriented’ issues; physician’s self-development; and respect for others | American Board of Internal Medicine (ABIM) (Lynch  et al. 2004; American Board of Internal Medicine 2005). |
|  |  |  | 32 | A 5-point Likert scale (1=Not important at all, 5= Very important) | Self administration | Integrity, social responsibility, professional practice habits, enduring quality care, altruism, and self-awareness. |  |
| Blue’s Multiple instruments [51] | 666 | Matriculating medical students’ knowledge and attitudes towards professionalism | Attitude instruments: 5 and 21; | First attitude instrument: 5 attributes on seven bipolar adjective scales (good-bad; fast-slow; small-large; pleasant-unpleasant; active-passive; awful-nice; light-heavy); the second attitude instrument: 5-point scale (strongly agree to strongly disagree)  First knowledge instrument: 15 vignettes to rank order from most professional to least professional action; second knowledge instrument: MCQ with true/false options | Self administration | Knowledge attributes: Humanism, Professional responsibility, Subordinating self-interest, Managing complexity and uncertainty, Professional commitment;  Attitude attributes: Humanistic values, Ethics and moral values, Self-reflection, Accountability, Subordinating self-interest | Swick’s (2000) [5] definition of professionalism and Vignettes were based upon the American Board of Internal Medicine vignettes |
| PSIQ [52] | 429 | Self-identity (professional identity formation) | 9 |  | Self administration | Interpersonal task, generic attributes, profession-specific elements |  |
| Hisar’s instrument for nursing students [53] | 1339 | Professionalism attitudes of nursing students in Turkey | 28 | A 5-point Likert scale (1=I do not agree at all, 5=I agree completely) | Self administration | Contribution to the increase of scientific information load; autonomy; cooperation; competence, continuous education; participation in professional organizations and professional development; working in committees; community service; ethical codes and theory | Literature review |
| Jiang’s knowledge instrument [54] | 1730 | First-semester medical students’ initial knowledge of important attributes of good doctors | 24 | A 5-point Likert scale | Self administration | Ethics, Skill, and Person | Qualitative study |
| LAMPS [55] | 413 | Attitudes of medical students on professionalism in the Arabian context | 28 | A 5-point Likert scale (1=strongly disagree, 5=strongly agree) | Self administration | Duty/Accountability, Excellence/ Autonomy, Honor/ Integrity, Altruism, Respect | ABIM |
| Wittich Reflection instrument [56] | 161 | Medical Students’  Critical Reflections on Professionalism | 12 | A 5-point Likert scale (1=disagree, 5=agree) | Self administration | Minimal reflection (Habitual action and understanding), moderate reflection (understanding and reflection), High reflection (critical reflection) | Based on the previously validated instrument [118, 119] |
| The new PAS[57] | 112 | Students’ perceptions of and attitudes towards professionalism in medicine | 22 | A 5-point Likert scale (1=strongly disagree, 5=strongly agree) | Self administration | Empathy and humanism,  Professional relationship and development, and Responsibility | Focus group discussion and Delphi method |
| DUQuE professionalism instrument [58] | 4872 | Physicians’ and nurses’ commitment to professionalism | 23 | Attitude item (15): A 5-point Likert scale (1=strongly disagree, 5=strongly agree); behavior items (8): Yes or No | Self administration | Attitude aspect: Improving quality of care, maintaining professional competence, fulfilling professional responsibilities, interprofessional collaboration. Behaviors aspect: Professional Quality Improvement Actions | ABIM and Code of Ethics for Nurses [120] |
| **Simulation (OSCE)** | | | | | | | |
| ECFMG^®^-CSA^®^ [59] | 7746 | Professionalism behavior | 10 SPs | A 4-point Likert scale (1=poor, 4=excellent) | SPs assess the candidates (OSCE) | Skills in interviewing and collecting information, Skills in counseling and delivering information, Rapport, Attentiveness, Personal manner | Professionalism as defined by the ABIM and the AAMC Medical School Objectives Project (MSOP) |
| *p*-OSCE [60] | 189 | Professionalism behavior | 6 stations | 3=pass; 2=borderline; 1=fail | SPs assess the candidates (OSCE) | Altruism, trust, and patient interest; Patient autonomy; Social justice | ACGME |
| **Multi Source Feedback** | | | | | | | |
| GMC patient and colleague questionnaire [39] | 18944 | Professional performance of doctors | 18 for patient survey, 25 for colleague survey | 9 for patient survey and 17 for colleague survey is 5-point Likert scale (1=poor, 5=strongly agree); 2 for patient survey and 1 for colleague survey is binary (yes/no) | Patient and colleague assess physicians in real situation/ 0.75 (36) for patient; 0.76 (12) for colleague |  | Good medical practice, which is the GMC’s core guidance on the principles and values to which it requires registered doctors to adhere |
| *p*-360°evaluation [60] | 189 | Professionalism behavior | 6 | 3=pass; 2=borderline; 1=fail | One nurse, two physicians (one attending physician and one chief physician), one CR, and one patient for each residents | Altruism, trust, and patient interest; Patient autonomy; Social justice | ACGME |
| **Direct observation** | | | | | | | |
| UMDSPAI[61] | 148 | Specific aspects performance of professionalism in surgical residents | 15 | A 5-point Likert-type scale: each score represents difference professionalism expression | Faculty assess residents and residents’ self-evaluation in real situation |  | ACGME competencies |
| P-MEX[62-64] | 1232 | Professionalism behavior in medical practice | 24 | A 4-point Likert scale (1=unacceptable; 4=exceeded expectations; 5= not observed or not applicable) | Evaluators assess the residents’ performance in in any situation where a student’s behavior can be observed / 0.8 (11), 0.81 (16), 0.8 (18), respectively. | Doctor-patient relationship skills, reflective skills, time management, and interprofessional relationship skills | Literature review and experts committee |
| EPRO-GP instrument[38] | 12 | Professional behavior in general practice | 127 | A 4-point Likert scale (1=hardly never, 4= almost always) | Self assessment and trainers’ assessment in real situation | Professionalism towards the patient; other professionals; the public; and oneself. | Literature review and committee meeting |
| Nijmegen Professionalism Scale [65] | 232 | Professional behavior of general practitioner | 106 | A 4-point Likert scale (1=never, 4=always) | GP trainees every 3 months assess trainees, and the GP trainees assess themselves in real situation | Professionalism towards the patient (respecting patient’s interests; and professional distance), professionalism towards other professionals (collaboration skills; and management skills.), professionalism towards society (responsibility; and quality management.), and professionalism towards oneself (reflection and learning; and dealing with emotions.). | The evaluation of professional behavior in general practice (EPRO-GP) instrument [68] |
| Adaptation of AACS fro foreigner [66] | 54 | Professional behavior of foreigner medical graduates | 25 | A scale with scores ranges form 4 to 10 (7=being the expected level of performance) | Supervisor, residents, and nurses assess the graduates | Courteousness, Communication, Collaboration, Dedication, Self-appraisal | Literature review and expert committee meeting |
| *p*-mini-CEX [60] | 189 | Professionalism behavior | 6 | 3=pass; 2=borderline; 1=fail | One attending  Physician assess residents | Responsibility to patients, Respect for patients | ACGME |
| **Peer assessment** | | | | | | | |
| Cottrell’s peer assessment [67] | 111 | Professionalism performance | 9 | A 7-point Likert scale (1=too little, 4=too much) and “0” for not observe | Medical students assess medical students; Generalizability Coefficients=0.7 for 13 raters | Honesty and Integrity, Accountability, Responsibility, Respectful and Nonjudgmental Behavior, Compassion and Empathy, Maturity, Skillful Communication, Confidentiality and Privacy in all patient affairs, Self-directed learning and appraisal skills | Swick HM’s definition of the construct of professionalism and 【】 code was developed by the medical school curriculum committee |
| **Patients’ opinion** | | | | | | | |
| Chandratilake’s general public scale [68] | 953 | Importance placed by the general public on doctors’ professional attributes | 55 | A 5-point Likert scale (1=unimportant, 5=extremely important) | Patient Self-administration | Interaction with patients, interaction with co-workers and the work place, and interaction with society | General Medical Council and ABIM |
| **Role model evaluation** | | | | | | | |
| Ephgrave’s Assessment[69] | 69 | Professionalism behavior of faculty in resident’s view | 20 | A 7-point Likert scale (1=never, 7=always) | Residents assessed faculties | Altruism; honor and integrity; respect; caring, compassion, and communication; and responsibility and accountability | Literature review |
| Arnold’s scale-environment version [70] | 404 | Measuring professionalism in the clinical environment in a comprehensive fashion | 12 | A 4-point Likert scale (1=never, 4=always) | Students to assess peers’, residents’, and faculty’s behaviors and faculty’s teaching; faculty self-assess their teaching |  | ABIM |
| LEP survey[71] | 903 | Medical student perceptions of professionalism among residents and faculty during clinical rotations | 11 | A 4-point Likert scale (4=consistently, 1=never;) | Self administration, (students assess residents and attending physicians) | Professional behavior and unprofessional behavior | ABIM |
| PACT[72] | 4715 | Professional and clinical teaching behaviors of clinical teachers | 17 | A 7-point Likert scale (1=Unsatisfactory, 7=Excellent) | Clerk assess their supervisor/ 0.8 (24) | Clinical skills, teaching skills, modeling the healer role, modeling the professional role | Original PACT instrument [121] |
| **Professionalism environment** | | | | | | | |
| PEFWQ® [73] | 848 | Key professionalism attributes and key environmental attributes that influence the professionalism of nurses in practice environments | 82 | A 5-point Likert Scale (from 1 =strongly agree, 5=strongly disagree) | Self-administration | Commitment, Collaborative practice, Advocacy, Accountability, Knowledge, Competence, Professional support, Shared governance, Control of nursing practice, Autonomy, Professionhood, Environment culture and climate, Quality of nursing work life | Literature review |
| Gillespie’s scale [74] | 85 | Residents’ Perceptions of Their Own Professionalism and the Professionalism of Their Learning Environment | Perceived professionalism survey: 20; learning environment survey: 11 | Perceived professionalism: a 4-point Likert scale (1=rare, 4=all of the time; learning environment: a 5-point Likert scale (1=not at all, 5=6 or more times) | Self-administration | Accountability to Patients, Society and the Profession (both); Commitment to Ethical Principles (both); Responsiveness to the Needs of Patients and Society that Supersedes Self-interest (Altruism); Commitment to Excellence and Ongoing Professional Development (both); Demonstrate Respect, Compassion, and Integrity (both); Demonstrate Sensitivity and Responsiveness to Patients | ACGME, American Board of Medical Specialties, ABIM, and Association of American Medical Colleges definitions of professionalism |
| **Professionalism as a facet of competency** | | | | | | | |
| **Self-administered rating** | | | | | | | |
| Hotjat’s Jefferson competency scale[75] | 4560 | Competence in patient care | 24 | A 4-point Likert scale (4=top quarter, 1=bottom quarter) and a option: insufficient information to judge | Self administration | Knowledge and Clinical Capabilities, Professionalism |  |
| ABIM Patient Assessment-self assessment version [76] | 130 | Residents’ communication skills and professionalism in dealing with patients | 11 | A 5-point Likert scale (1=poor, 5=excellent) | Self administration | Interpersonal relations and conveying medical information | The ABIM's Patient Assessment survey |
| NPVS-R [77, 78] | 782 | Nurses’ professional values | 26 (4) | A 5-point Likert scale (1=not important, 5=most important) | Self administration | Caring, Activism, Trust, Professionalism, and Justice | Code of Ethics for Nurses [120] |
|  | 286 | Nurses’ professional values | 12 (13) | A 5-point Likert scale (1=not important, 5=most important) | Self administration | Professionalism, Caring, Activism, | Code of Ethics for Nurses [120] |
| VPPVS [79] | 1086 | Physicians’ professional values | 35 (8) | A 5-point Likert scale (1=strongly disagree, 5=strongly agree) | Self administration | Lifestyle, Prestige, Professionalism, management, financial | Previous instrument (physician values in practice scale) [123] |
| NPRCS [80] | 563 | Roles and competencies of nurse practitioners | 54 (20) | A 5-point Likert scale (1=completely disagree, 5= completely agree) | Self administration | Professionalism, direct care, clinical research, practical guidance, medical assistance, leadership and reform | Previous instrument [124, 125] |
| **Multi Source Feedback** | | | | | | | |
| Musick 360-degree instrument [81] | 421 | Clinical performance and professionalism of residents | 26 (9) | A 9-point Likert scale (1=unsatisfactory performance, 9=outstanding performance) | 360-degree evaluation | Quality of patient care, Personal characteristics/ Professionalism, Communication | Literature review and expert assessment |
| Wood’s 360-degree evaluation [82] | 57 | Competence in professionalism and interpersonal/communication skills of Radiology resident | 10 | A 5-point Likert scale (1=strongly disagree; 5=strongly agree) | 360-degree evaluation |  | Education Committee of the Association of Program Directors in Radiology’s six competencies for radiology residency [126] |
| CPSA-PAR MSF for anesthesiologists [83] | 6143 | Feedback to physicians about a broad range of competencies | Patients: 11; coworkers: 19; medical colleagues and self: 29 | A 5-point Likert scale for patients (1=strongly disagree, 5=strongly agree); a 5-point Likert-type scale for medical colleagues, co-workers, and self (1=among the worst, 5= among the best) | 360-degree evaluation/ 0.65 (17.7) for patients, 0.56 (7.75) for coworkers and 0.69 (7.82) for colleagues | Patient: professionalism and communication; coworkers: communication and collaboration; medical colleague and self: clinical performance, communication and professionalism, and continuing professional development | Literature review previous instruments |
| CPSA-PAR MSF for emergency physicians[84] | 7076 |  | Patients: 16; coworkers: 20; medical colleagues: 30; and self: 31 | A 5-point Likert scale for patients (1=strongly disagree, 5=strongly agree); a 5-point Likert-type scale for medical colleagues, co-workers, and self (1=among the worst, 5= among the best) | 360-degree evaluation/ 0.68 (25) for patients, 0.85 (8) for coworkers and 0.84 (8) for colleagues | Patient: communication/professionalism and patient education; coworker: communication/collegiality and professionalism; medical colleague and self: clinical performance, professionalism. Self-management, and record keeping. | Previous instruments and the CanMEDS’s competencies |
| CPSA-PAR MSF for pediatricians [85] | 3963 |  | Patients: 40; coworkers: 22; medical colleagues: 38; and self: 37 | A 5-point Likert scale for patients (1=strongly disagree, 5=strongly agree); a 5-point Likert-type scale for medical colleagues, co-workers, and self (1=among the worst, 5= among the best) | 360-degree evaluation/ 0.85 (23.4) for patients, 0.87 (7.6) for co-worker. 0.78 (7.6) for medical colleagues | Patient: patient care, technical communication, staff, and office function; coworker: humanistic and psychosocial skills, collegiality, and written communication; medical colleague and self: patient management, clinical assessment, professional development, and communication skills | Previous instruments and the CanMEDS’s competencies |
| CPSA-PAR MSF for international doctors [86] | 1130 |  | Patients: 13; coworkers: 13; medical colleagues: 22; and self: 21 | A 6-point Likert scale (0= unable to assess, 1=strongly disagree, 5=strongly agree) | 360 degree evaluation/ 0.71 (17.5) for patient, 0.59 (6.9) for co-worker, 0.76 (6) for medical colleagues | Patient survey: professionalism and communication skills, and system management; coworker survey: professionalism and communication; medical colleague survey: clinical care and professionalism | Literature review and committee meeting |
| CPSA-PAR MSF for Psychiatrists[87] | 4065 |  | Patients: 40; coworkers: 22; medical colleagues: 38; and self: 37 | A 5-point Likert scale for patients (1=strongly disagree, 5=strongly agree) | 360 degree evaluation/ 0.78 (24.32) for patient; 0.82 (7.37) for coworker, and 0.81 (7.56) for medical colleagues | Patient: Communication skills, professionalism, collegiality, and self-management; coworker: humanistic and psychosocial, coworker collegiality, and written communication: medical colleague: humanistic and communication, psychosocial management of patients, clinical performance, and professional self-management |  |
| CPSA-PAR MSF for physicians [88] | 17446 |  | Colleague: 31; co-workers: 17; patient: 40 | A 5-point Likert scale for patients (1=strongly disagree, 5=strongly agree); a 5-point Likert-type scale for medical colleagues, co-workers, and self (1=among the worst, 5= among the best) | 360 degree evaluation/ 0.81 (10) for colleague; 0.86 (10) co-workers; 0.80 (23) patient | Colleagues: professionalism, clinical competency, and psychosocial management; co-worker: professionalism and communication; patients: professionalism and communication; office personnel; access to doctor; physical space |  |
| CPSA-PAR MSF for Pathologists/Laboratory Medicine Physicians [89] | 2356 |  | Peer and self: 39; physicians: 30: coworker survey: 22 | A 5-point Likert scale (1=strongly disagree, 5=strongly agree) and an unable to assess category | 360 degree evaluation/ 0.78 (8) for peer, 0.81 (8) for referring physicians, and 0.81 (8) for coworker | Clinical competence, collaboration, professionalism, and communication for the self; professionalism and communication for the coworker; professionalism, reports, and clinical competency for the referring physicians, and reports and clinical competency collaboration, educational leadership, and professional behavior for peer |  |
| CPSA-PAR MSF for Middle eastern interns [90] | 335 |  | 39 (15) | A 5-point Likert scale (1=among the worst, 5=among the best) | Peer assessment/ 0.81 (10)-all together | Professionalism, communication skills, and collaboration | Expert committee |
| End-of-rotation evaluations [91] | 4986 | Behavior of learners in workpalce |  | A 9-point Likert scale (1-3: Unsatisfactory; 4-6 satisfactory: 7-9:superior) | Residents were assessed by fellows, faculties and program directors during the rotation/ 0.7(14 evaluations) | ACGME core competencies (interpersonaland communication skills, medical knowledge, patient care, practice-based learning and improvement, professionalism, systems-based practices) | ACGME core competencies |
| EOS group 360-degree instrument[92-94] | 148 | Resident doctors’ competency in professionalism and communication skills | Office staff survey 15; patient survey 14; Nurses survey 26; peer, self, and attending’s survey 21; | A 5-point Likert scale (1=never, 5=always) and an unable to assess category | 360 degree evaluation | Professionalism and interpersonal and communication skills for office staff, Nurses, peers, attending physicians, and self; patient care, professionalism, interpersonal & communication skills, and systems based practice | ACGME |
| Senol’s Turkish 360-degree assessment [95] | 6630 | Performance of medical practitioners from different sources that have different type of relations with them | Peer survey: 38, nurse survey: 33, auxiliary survey 7, secretary survey 15, patient survey 9 and self survey 58 | A 5-point Likert scale (1=never, 5=always) | 360 degree evaluation | Patient care, medical knowledge, practice-based learning, professionalism, communication skills, and systems based practice | ACGME and literature review |
| Overeem’s MSF instruments [96] | 3864 | Physicians’ professional performance in actual clinical practice | Peer survey: 33: co-worker survey: 22; and patient: 18 | A 9-point Likert type scale: (1 = completely disagree, 5 = neutral, 9 = completely agree). | 360 degree evaluation/ 0.8: 5 for peers; 6 fro coworkers; and 12 for patients | Peer survey: Collaboration, Clinical performance, Practice based learning and improvement, Coordination and continuity, Responsibility and time-management, Emergency medicine; coworker survey: Relationship with healthcare professionals, Communications with patients, and Professionalism; patient survey: patient-centeredness | Experts committee previously developed MSF instruments for medical and surgical specialties in Canada owned by the College of Physicians & Surgeons of  Alberta [127] |
| **Direct observation** | | | | | | | |
| ACGME-TRF [97] | 343 | ACGME’s six competency of residents | 25 (7) | A 3-point Likert scale (1= above average, 3= required counseling for significant deficiencies) | Global view of supervisor | Professionalism, patient care, medical knowledge, and communication | ACGME competencies |
| Global rating form for ACGME competencies [98] | 1295 | ACGME’s six competency of residents | 23 (7) | A 5-point Likert scale (1=severe deficits, 5=outstanding competence) | Directors of residency program assess residents | Interpersonal communications skills and professionalism; Systems based practice, and practice-based learning and improvement. | ACGME competencies |
| OCEX[100, 101] | 112 | Core residency competencies identified by the ACGME | 33 (7) | A 5-point Likert scale (1=Does not meet expectation, 2=meets some expectation, 3=meets all expectation, 4=exceeds expectation) | Directors of residency program assess ophthalmic residents | Interview skill, examination, interpersonal skills/ professionalism, case presentation | ACGME competencies |
| ACGME general competencies [99] | 150 | ACGME’s six competency of emergency medicine residents | 61 | A 9-point Likert scale | Directors of residency program assess residents | Patient care, Medical knowledge, Practice-based learning and improvement, Interpersonal and communication skills, Professionalism, Systems-based practice | ACGME competencies |
| Durning’s Supervisor’s evaluation form [102] | 1247 | An outcome measurement for our undergraduate medical education institution | 18 (6) | A 6-point Likert scale (0= unable to judge, 1=not satisfactory, 5=outstanding) | Supervisors assess residents | Professionalism, and expertise | Experts committee meeting |
| Durning’s Supervisor’s evaluation form-PGY3[103] | 388 |  | 58 (9) |  | Supervisors assess residents | Medical Expertise; Professionalism;  Military-unique Practice; Systems-based Practice | ACGME |
| Karayurt nursing students’ performance [104] | 97 | Nursing students’ performance | 26 (9) | A 10-point Liker type scale (1=severe deficits, 10=outstanding competence) | Supervisors assess nursing students | Nursing process, Professionalism, Ethical principles | Literature review and Experts committee meeting |
| COMPASS [105] | 184 | Monitor performance of residents in practice throughout the entire training | 17 (3) | A 9-point Liker type scale (1=extremely unsuitable, 9=extremely suitable) | GP raters assess GPs depending on their performance in last three months | Medical expertise, Communication, Management, Collaboration, Social Accountability, Science and Education, and Professionalism | CanMEDs role models |
| Handoff CEX[106, 107] | 771 | An opportunity for critical reassessment and error reduction of performance in practice | Handoff provider: 6 (1); Handoff recipient: 5(1) | A 9-point Likert scale (1-3: Unsatisfactory; 4-6 satisfactory: 7-9:superior) | Peer assessment by real-time observation | Setting, organization, communication, content, judgment and professionalism | ACGME |
| ITER [108] | 172 | Key competencies of residents as mandated by CanMEDS roles | 24 (5) | A 5-point Likert scale (1=fails to meet, 5=exceeds expectations) | ER clinical evaluators assess residents by ITER at the end of rotation | Medical Expert, Communicator and Collaborator, Professional, Health Advocate, and Manager | CanMEDS |
| Dong’s Graduates Form [109] | 293 | medical graduates’ first-year internship performance | 17 (7) | A 4-point Likert scal | Resident program director assess residents | Medical Expertise, Military-unique Practice, Professionalism, System-based Practice, and Communication and Interpersonal Skills | ACGME competencies |
| **Simulation (OSCE)** | | | | | | | |
| Standardized Direct Observation Assessment Tool (SDOT) [110] | 82 | ACGME Core Competency | Two videos of simulated patient–resident–attending physician encounters and 26 expected behaviors | Needs improvement, meets expectations, or above expectations | Faculty assess the performance of residents | Patient care, Medical knowledge, Practice-based learning and improvement, Interpersonal and communication skills, Professionalism, Systems-based practice | ACGME competencies |
| Jefferies’s OSCE of CanMEDS Roles [111] | 24 | CanMEDS 7 physicians’ competencies | 10 stations, 10 item of checklist | A 5-point Likert scale (each score means different level of performance) | OSCE | Medical expert, communicator, collaborator, manager, health advocate, scholar, professional | CanMEDS Roles |
| Ponton-Carss Checklist of OSPRE [112] | 89 | One part of the whole competency (derived from competency models) | 24 (6) | A 5-point Likert scale (1=poor, 5=excellent) | Raters assess the residents during the OSCE/OSPRE by the checklist | Communication skills, professionalism, and surgical skills | CanMEDs role models, Objective Structured Assessment of Technical Skill (OSATS) [129, 130]and Jefferies’s OSCE of CanMEDS Roles professionalism checklist [110] |
| RO&CA [113] | 362 | Specific competencies, and immediate feedback to residents | 9 (2) | A 4-point Likert scale (1=unsatisfactory, 4=superior) | Attending physicians assess residents by 10-15 min observe patient-resident interaction | Patient care, professionalism, interpersonal and communication skills, system-based practice, practice based learning and improvement | Review of the existing mini-CEX format |
| ACGME competency checklist of OSCE[114] | 192 | Residents’ acquisition of the six core competencies | 12 (2) | Dichotomous measure of 0 and 1 (1=presented, 0= not presented) | Raters assess the residents during the OSCE/OSPRE by the checklist | Patient care skills; interpersonal and communication skills; professionalism skills; practice-based learning and improvement skills; systems-based practice skills; medical knowledge skills | ACGME competencies |
| CanMEDS OSCE [115] | 150 | Multiple intrinsic CanMEDS roles and performance | 29 (5) | A 5-point Likert scale (1=needs significant improvement, 5=superb) | OSCE | Communicator, Collaborator, Manager, Health advocate, Scholar, Professional | CanMEDS |
| **Role model evaluation** | | | | | | | |
| Smith’s instrument [120] | 731 | The performance of inpatient teaching attending physicians | 32 (5) | A 5-point Likert scale (1=never, 5=always) | Residents assess attending physicians | Evidence-based medicine, bedside teaching, clinical reasoning, patient-based teaching, teaching sessions, patient care, rounding, professionalism, and feedback. | Literature review and committee meeting |
| Faculty Supervision Evaluation [116] | 19 residents, 39 instructors | Faculties’ supervision performance | 9 (1) | A 4-point Likert scale | Residents assess their instructors; G coefficient=0.8 (4) | Providing feedback; being available; giving opportunities/fostering resident autonomy; stimulating patient-based learning; demonstrating professionalism; being present during the critical events; demonstrating interpersonal skills; being concerned about safety. | Experts committee meeting |
| Colletti evaluation of clinical educators [117] | 29 faculties | Faculties’ performance | 18 (10) | A 9-point Likert scale (1–3 =“below expectations”; 4–6 = “meets expectations”; and 7–9 = “exceeds expectations”.) | Residents assess their clinical educators | Competency and Professionalism, Commitment to Knowledge and Education, Inclusion and Interaction, Patient Focus, Openness and Enthusiasm | ACGME competencies |
| Perceived Faculty Competency Inventory (PFCI) [118] | 88 medical students | Faculty Competence | 33 (11) | 5-point Likert scale ranging from 1 (strongly disagree) to 5 (strongly agree) | Medical students assess faculty | Professionalism/Ethics; Clinical Supervision; Research; Multicultural Competence; Advising/Mentoring. | on the basis of accreditation, training, and curriculum  standards set forth by the American Psychological Association  (APA, 2009) |
| **Professionalism environment** | | | | | | | |
| MSSAPS [119] | 228 | Medical students’ perceptions of safety culture, teamwork culture, error disclosure culture, and professionalism | 39 | A 5-point Likert scale (1=strongly disagree, 5=strongly agree) | Self administration | Safety culture, Teamwork culture, Error disclosure culture, Experiences with professionalism, Comfort expressing professional concerns | Previous related instruments |

* How many surveys were required to obtain an equivalent dependability coefficient. Numbers outside the parentheses are the dependability coefficient. Numbers inside the parentheses are how many survey required.
